# The prevalence of long-term neurodevelopmental outcomes in preterm-born children in low- and middle-income countries: a systematic review and meta-analysis of developmental outcomes in 72 974 preterm-born children

**DOI:** 10.7189/jogh.15.04106

**Published:** 2025-04-04

**Authors:** Saima Sultana, Sayaka Horiuchi, Caroline SE Homer, Abdullah H Baqui, Joshua P Vogel

**Affiliations:** 1 Monash University, School of Public Health and Preventive Medicine, Melbourne, Australia; 2 Burnet Institute, Women’s, Children's and Adolescents’ Health Program, Melbourne, Australia; 3 Japan Society for the Promotion of Science, Overseas Research Fellowship, Tokyo, Japan; 4 Johns Hopkins Bloomberg School of Public Health, Department of International Health, Baltimore, Maryland, USA

## Abstract

**Background:**

Preterm birth is associated with an increased risk of adverse neurodevelopmental outcomes. However, prevalence estimates of adverse neurodevelopmental outcomes on preterm born children in low – and middle – income countries (LMICs) remain unclear. In this systematic review and meta-analysis, we aim to estimate the prevalence of adverse neurodevelopmental outcomes in preterm-born children in LMICs.

**Methods:**

We comprehensively searched six electronic databases – Medline, Embase, CINAHL, PsycInfo, Scopus, and Web of Science, without language and date restrictions. We included observational studies conducted in LMICs that reported prevalence of any type of neurodevelopmental outcome in children born preterm using a validated method or clinical diagnosis, and outcome measurement was performed in at least 100 eligible children at age ≥12 months. The primary outcomes of interest were a composite of any neurodevelopmental impairment, cerebral palsy, visual impairment/blindness, hearing impairment/deafness, motor impairment, developmental delays, learning difficulties, and adverse behavioural and socio-emotional outcomes. We used the JBI critical appraisal checklist to assess the quality of the included studies, and prevalence estimates were calculated using a random-effects meta-analysis model.

**Results:**

A total of 47 data sets from 12 countries involving 72 974 preterm-born children were included. The estimated pooled prevalence of overall neurodevelopmental impairment and cerebral palsy was 16% (95% confidence interval (CI) = 11–21%) and 5% (95% CI = 3–6%), respectively. The pooled prevalence of developmental delays across different domains ranged from 8 to 13%. Lower prevalence was found in hearing impairment/deafness and visual impairment/blindness (1%). Higher prevalences were observed with decreasing gestational age and birth weight.

**Conclusions:**

There is a high burden of adverse neurodevelopmental outcomes in preterm born children in LMICs. Such prevalence estimates are essential in informing clinical and public health policy, allocating scarce resources, and directing further research to improved outcomes in these settings.

**Registration:**

PROSPERO: CRD42024569564.

The World Health Organization (WHO) defines preterm birth as occurring before 37 completed weeks of gestation or fewer than 259 days since the first day of a woman's last menstrual period [1]. Globally, an estimated 13.4 million neonates are born preterm each year, with over 80% of these births occurring in sub-Saharan Africa and South Asian countries [2,3]. Complications related to preterm birth are the leading cause of death in newborns and children younger than five years of age; preterm birth accounts for approximately one million child deaths each year [4]. Preterm birth is also associated with a heightened risk of long-term morbidities, including adverse neurodevelopmental outcomes, such as, neurosensory impairments, behavioural and emotional problems, cognitive deficits and learning difficulties. These risks escalate at lower gestational ages and birth weights [5–10].

While some adverse preterm outcomes are identified in the first two to three years of life, some behavioural and socio-emotional problems only become evident in schooling years [11]. With advancements in perinatal and neonatal care practices – such as antenatal corticosteroids, surfactant and non-invasive ventilation – the survival rate of preterm infants has increased over the past few decades [12]. However, improved survival means that rates of impaired neurodevelopment in childhood is probably rising. Long-term sequalae of preterm birth negatively impacts academic attainment, employment and quality of life [10]. Until recently, little attention has been given to these longer-term outcomes in preterm born children in low and middle-income countries (LMICs).

Neurodevelopmental trajectories are dynamic and influence various developmental stages. Early detection, timely intervention, and access to rehabilitation services are crucial to maximising children’s developmental potential, improving overall functioning outcomes, and mitigating ongoing health risks [13]. While a number of effective options exist for children with neurodevelopmental disabilities, these services are not available in most LMICs – indicating the substantial unmet needs of children and adolescents with disabilities [14]. Furthermore, existing services are often fragmented, underfunded, of subpar quality, costly, and primarily concentrated in urban areas [14,15]. While limited resources are a key barrier to implementing these services in LMICs, lack of data on prevalence estimates, including unmet needs often hinders effective policy making and programme implementation. Considering the majority of the world’s preterm births occur in LMICs, it is crucial to estimate the magnitude of the longer-term outcomes in these countries, particularly adverse neurodevelopmental outcomes. Such data would help researchers, health care providers and policymakers to better understand the burden of these conditions, identify priority areas, and thus design and implement appropriate clinical and public health interventions.

While a number of LMIC-based studies have explored specific neurodevelopmental outcomes, these have not been pooled. A few systematic reviews have assessed the prevalence of individual conditions or focused on outcomes for selected subpopulations *i.e*. extremely low gestational age or very low birth weight infants [16,17]. To fill this knowledge gap, this systematic review and meta-analysis aims to estimate the prevalence of adverse neurodevelopmental outcomes in preterm-born children in LMICs.

## METHODS

We conducted this systematic review following the Preferred Items for Systematic Reviews and Meta-Analysis (PRISMA) guidelines [18]. Our protocol for this systematic review was registered on PROSPERO (CRD42024569564).

### Data sources and search strategy

In consultation with an experienced academic librarian, we developed a comprehensive search strategy and searched Medline, Embase, CINAHL, PsycInfo, Scopus, and Web of Science. The search strategy included keywords and subject headings relevant to the population and outcomes. There were no limits on the year of publication and language. In addition, we searched references cited in the included articles to avoid missing relevant articles. The initial search was conducted on 20 March 2023, and updated on 23 April 2024. The full search strategy is available in Appendix S1 in the **Online Supplementary Document**.

### Selection criteria

We included studies if they reported prevalence of any type of neurodevelopmental outcome in children born preterm (*i.e*. birth <37 weeks of gestation) through clinical diagnosis or using a validated diagnostic/screening measurement tool or reported data from which such prevalence can be derived. Only studies that measured the outcomes in at least 100 eligible children at age ≥12 months were included. A pragmatic, minimum sample size of 100 was used for inclusion to ensure the precision and reliability of prevalence estimates, while also minimising potential bias. We included observational studies, particularly cross-sectional studies, retrospective cohort or prospective cohort studies, that were conducted in a low, lower-middle, or upper-middle income country as defined by the World Bank [19]. We excluded studies if the study participants were term-born children or are adults (older than 18 years) or a population comprised only of preterm babies with other, less common complications (*e.g*. short bowel syndrome, microcephaly, foetal inflammatory response syndrome). We also excluded reviews, editorials, interventional studies, case-control studies, case series and case reports, and conference abstracts.

The primary outcomes for this review were neurodevelopmental outcomes assessed at ≥12 months after birth: a composite of any neurodevelopmental impairment; cerebral palsy; visual impairment/blindness; hearing impairment/deafness; motor disorders; developmental delays subdivided into: cognitive delay, motor delay, language delay, and global developmental delay; learning difficulties, and adverse behavioural and socio-emotional outcomes. The secondary outcome was growth outcomes, such as underweight and stunting. Operational definitions of neurodevelopmental outcomes used in this review are available in Appendix S2 in the **Online Supplementary Document**.

Two independent reviewers (SS, SH) screened the titles/abstracts and potentially eligible full texts following the eligibility criteria. Any disagreement was resolved by consensus, or a third reviewer was consulted. We used Google Translate to translate non-English articles.

### Data extraction and study quality assessment

The same two reviewers independently extracted data using an Excel spreadsheet. Any disagreement or uncertainty was resolved by discussion. We extracted data on: author and year of publication, country of study, country income status, study design, study population characteristics (birth year, gestational age, birthweight, age at follow up), author defined outcome definitions, methods and tools used to assess the outcomes, total number of eligible participants, and number of participants with reported outcomes. The two reviewers independently assessed the quality of included studies using the JBI critical appraisal checklist for prevalence data, and studies were rated as high quality (7–9), moderate quality (4–6), or low quality (0–3) [20,21]. Any disagreements were resolved by consensus or by consulting a third reviewer.

### Data synthesis

Data analyses used STATA, version 18 (StataCorp LLC, College Station, Texas, USA). Pooled prevalence estimates were calculated using a random-effects meta-analysis with the Freeman-Tukey double arcsine transformation [22]. Due to the anticipated heterogeneity, a random effects model was used, as it provides higher generalisability under heterogenous conditions compared to a fixed effects model [23]. Forest plots were used to visualise the pooled estimates. Heterogeneity between studies was assessed using Cochran’s Q and *I*^2^ statistic, with an *I*^2^ of more than 50% indicating substantial heterogeneity. Potential sources of heterogeneity were investigated by subgroup analyses, stratifying studies by gestational age, birthweight, and country-income level. We classified studies into one of three gestational age subgroups (<28 weeks; 28 to <32 weeks; 32 to 36 weeks) based on the mean gestational age of the study population, and also into three birth weight subgroups (<1500g; 1500 to <2500g; ≥2500g) using the mean birthweight of the study population. We also performed a ‘leave one out’ sensitivity analysis to identify what (if any) outliers impacted overall prevalence estimates [24]. We also performed sensitivity analyses by excluding studies categorised as low or moderate quality. The possibility of publication bias was examined using funnel plots and Egger’s test.

## RESULTS

**Figure 1:**
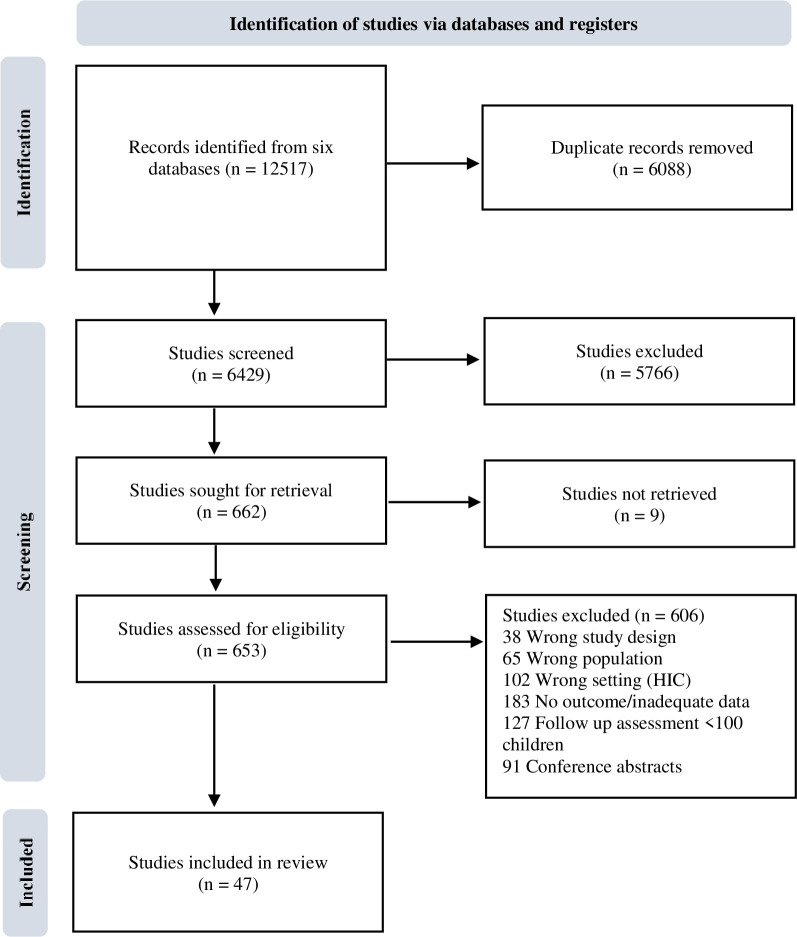
PRISMA Flow Diagram.

Our search identified 6429 citations. After title and abstract screening, 663 full texts were assessed for eligibility and 47 met the inclusion criteria (**Figure 1**). Of these, five publications used data derived from two unique birth cohorts, creating 47 data sets from 44 studies.

The included studies reported on the outcomes of 72 974 preterm-born children from 12 countries, predominantly upper-middle-income countries (**Table 1**). All studies were published between 1992 and 2023. The majority of studies included were conducted in China (n = 22, 50%), followed by Brazil (n = 7, 11%). Most studies had a mixed population, including children with and without complications related to preterm birth. However, one study specifically focused on children with leukomalacia [30]. Gestational age at birth ranged from 22 weeks to <37 weeks. Eligible studies performed developmental assessments in children between the ages 12 months and 12 years. Using the JBI critical appraisal tool, 28 (59.6%) studies attained a score of 7 or higher (high quality), while 19 (40.4%) studies scored between 4 and 6 (moderate quality), and no studies rated as low quality.

**Table 1:** Study characteristics

Study ID	Study design	Country	Country income category	Study period	Population	Birth year	GA at birth (wks) x̄ (SD)/range	Birth weight (gm)x̄ (SD)/range	Age at follow up	Sample size (preterm population)	Method/measurement tool for neurodevelopmental assessment	JBI tool quality assessment score
Liu 2023 [25]	Cross-sectional	China	Upper middle	Not available	Children aged 3–10 y	Not available	<37	Not available	3–10 y	156	MABC-2	8
Bao 2023 [26]	Cohort	China	Upper middle	2021–2023	Infants aged 12 to <13 mo who attended the Outpatient Service of Child Care in the First Affiliated Hospital of Shandong First Medical University	Not reported	<37	2716.9	12–<13 mo	144	NDSC	4
Saha 2023 [27]	Retrospective cohort	India	Lower middle	2013–2015	Late preterm infants, born at gestational age between 34 0 / 7 and 36 6 / 7 weeks admitted to neonatal intensive care unit, and followed up at neurodevelopment clinic	2013–2015	34.2 (0.82)	1719	2 y CA	299	Clinical/neurological, visual and hearing examination, assessment, DASII, anthropometry	7
Metwally 2023a [28]	Cross-sectional	Egypt	Lower middle	Dec 2017–Dec 2019	Children aged 1 to 12 y	Not reported	<37	Not available	1–12 y	413	M-CHAT, GARS-2	8
Metwally 2023b [29]	Cross-sectional	Egypt	Lower middle	Dec 2017–Dec 2019	6 to 12 y from 8 Governorates	Not reported	<37	Not available	6–12 y	186	VABS	8
Song 2023 [30]	Prospective cohort	China	Upper middle	Not available	Newborns diagnosed with leukomalacia	2015–2020	30.6 Range: 28.8–33.3	1613.7 (558.2)	2–7 y	114	GMDS-Chinese	8
Sivanandan 2022 [31]	Prospective cohort	India	Lower middle	Sep 2011–Nov 2015	Consecutively born preterm infants with gestational age ≤32 weeks or birth weight ≤1500 g	2011–2014	30.4 (2.0)	1294 (357)	18 mo CA	195	Clinical/neurological, visual and hearing examination, DASII, anthropometry	7
Ruan 2022 [32]	Cross-sectional	China	Upper middle	June–Dec 2019	Kindergarten children	Not available	<37	Not available	Not reported	4933	LDCDQ	6
Shen 2022 [33]	Cross-sectional	China	Upper middle	Nov–Dec 2005	Children aged 5–12 y studying in the selected schools	Not available	<37	Not available	6–12 y	1055	DSM-IV diagnostic criteria	7
Mackay 2022 [34]	Prospective cohort	South Africa	Upper middle	Jun 2017–Jan 2019	In-born infants with birth weight <1500g	2017–2019	30.3 (1.95)	1210.8 (187)	12 mo PMA	104	GMDS III	7
Wang 2022 [35]	Prospective cohort	China	Upper middle	Jul 2012 and Dec 2019	Preterm infants with a gestational age <30 weeks who were admitted to the NICU	2012–2019	28.6 (1.4)	1170 (300)	18–24 mo CA	915	BSID II	8
Ratanatharathorn 2022 [36]	Prospective study	Thailand	Upper middle	May 2017–Dec 2021	Premature infants (<37 weeks), who experienced either birth asphyxia or low birth weight (<2500 g)	2016–2020	30 (2.6)	1218 (352)	12 mo CA	126	BSID III	5
Hua 2022 [37]	Retrospective cohort study	China	Upper middle	2018–2019	Children with singleton delivery aged 3–5 y old from 2403 kindergartens in 551 cities	Not reported	<37	Not available	3–5 y	17 753	ASQ-3	8
Hua 2021 [38]	Retrospective cohort	China	Upper middle	Apr 2018–Dec 2019	Pre-schoolers children recruited from 2403 mainstream kindergartens in 551 cities	Not reported	<37	Not available	3–5 y	20 676	LDCDQ	8
Soldateli 2022 [39]	Cohort study	Brazil	Upper middle		Preterm infants participated in the 2004 Pelotas cohorts	2004	<37 weeks	2500 (600)	6 y	472	DAWBA, SDQ	7
Chen 2021 [40]	Retrospective cohort	China	Upper middle	2014–2019	Preterm infants with GA<32 weeks' gestation	2014–2019	29.7 (1.5)	1306 (295)	12 mo CA	1689	BSID-Chinese version	7
Do 2020 [41], Do 2021 [42]	Cohort follow up	Vietnam	Lower middle	Jul 2013–Sep 2014	Preterm newborns admitted to the NICU	2013–2014	31.6 (2.5)	1754 (484)	1 y and 2 y CA	184	BSID-III, clinical/neurological, visual and hearing examination, anthropometry	7
Du 2020 [43]	Cross-sectional	China	Upper middle	Not available	Children aged 3–10 y residing in urban China	Not available	<37	Not available	3–10y	2185	MABC-2 (Chinese)	8
Akshara 2020 [44]	Prospective study	India	Lower middle	Mar 2017–Jun 2018	Preterm babies (32–36 weeks) graduates from NICU	2017	32–36	Not available	12 mo CA	159	TDSC, DASII	5
Gonzalez-Andrade 2020 [45]	Cross-sectional	Ecuador	Upper middle	2015–2016	Preterm infants weighing <1500 g, admitted to the NICU or intermediate care with two or more comorbidities at birth	Not available	29–36	<1500 g	24–47 mo	138	Brunet-Lézine scale, Child Development Assessment test	6
Liu 2019 [46]	Cross-sectional	China	Upper middle	Jun–Aug 2017	Preterm infants between 1 and 3.5 y of age	Not available	<37	Not available	12 mo	309	Anthropometry	6
You 2019a [47]	Cross-sectional	China	Upper middle	2011–2013	Children born between 34 + 0 and 36 + 6 weeks of gestation	2011–2013	35.5 (1.02)	2796 (482)	24–30 mo	102	Clinical/neurological examination, GDDS-Chinese, M-CHAT, SIS	7
Li 2019 [48]	Retrospective study	China	Upper middle	Jan 2010–May 2016	Extreme preterm born infants (<28 weeks) admitted into 10 participating NICUs	2011–2016	22 to <28	≤1500	18–24 mo	131	Clinical/neurological examination, GDS, S-S relations	7
You 2019b [49]	Retrospective study	China	Upper middle	Not available	Children born with a GA between 34 + 0 w and 36 + 6weeks and were considered healthy and had no serious clinical issues post birth	2013–2015	35.02 (1.95)	2774 (469)	24–30 mo	112	Clinical/neurological examination, GDDS-Chinese version	6
Santos 2017 [50]	Population based birth cohort	Brazil	Upper middle	Multiple time point	All live births delivering at the maternity hospitals in Pelotas	2004	<37	Not available	6 y	416	DAWBA	8
Lin 2017 [51]	Population-based child cohort	China	Upper middle	2014 and 2015	Children at their first entrance into kindergartens in the Longhua District of Shenzhen	Not available	<36 weeks	Not available	1.3–5.7 y	1475	CPRS	6
Bozkurt 2017 [52]	Cross-sectional	Turkey	Upper middle	Not available	Preterm infants with a GA of ≤32 weeks who were admitted to NICU and survived to a corrected age of 18 to 22 mo	2008–2011	29.5 (1.9)	1267 (311)	18–22 mo CA	220	Clinical/neurological examination, BSID-II	6
Liu 2016 [53]	Cross-sectional	China	Upper middle	2011	Primary school students (grade 3 to 6) in seven cities of the Hubei province	Not available	<37	Not available	10.23 (1.22) y	2140	DCCC, PRS	5
Sujatha 2016 [54]	Prospective cohort	India	Lower middle	Jan 2005–Jul 2009	All preterm babies (≤33 wks gestation) discharged from NICU	2005–2009	30.6 (2)	Not available	1 y CA	225	Clinical/neurological, visual and hearing examination, DDST, DASII	7
Xue 2016 [55]	Cohort follow up	China	Upper middle	2008	Preterm infants discharged from the NICU and entered a 1-y follow-up programme	2012–2013	28.6 (2.3)	1361.9	1 y CA	105	Clinical/neurological examination, BSID-II	6
Baskabadi 2016 [56]	Cohort study	Iran	Lower middle	Not reported	Premature infants with history of NICU admission	Not available	31.8 normal dev: 32 (2.63); dev delay: 30.33 (2.82)	1563.8 g	12 mo and 24 mo	270	ASQ	6
Zhu 2015 [57]	Prospective study	China	Upper middle	Jan and Sep 2008	Babies born in the Hefei Maternal and Child Health Hospital	2008	<37 weeks	Not available	48–54 mo	105	CPRS	5
Moreira 2014 [58]	Cross-sectional	Brazil	Upper middle	Dec 2011–Jul 2012	Children who were born prematurely between 2002 and 2004, and were being followed at the Children at Risk Outpatient Clinic	2002–2004	<35; MD 31	MD 1370 g	8–10 y	100	MABC-2, TT, TDE	6
Xiong 2014 [59]	Cross-sectional	China	Upper middle	Jan–Mar 2011	School children aged 9–15 y	Not available	<37 weeks	Not available	9–15 y	226	Anthropometry	6
Ferreira 2014 [60]	Prospective study	Brazil	Upper middle	Not available	Preterm infants (<37 weeks) with birth weight less than 1500 g	2004–2010	29.9 (2)	1119 (247)	12 mo CA	194	Clinical/neurological examination, BSID-II	8
Eras 2013 [61]	Prospective study	Turkey	Upper middle	Not available	Preterm infants (≤32 weeks) born and hospitalised at neonatal intensive care unit	2008–2009	29.0 (2.3)	1247.7 g; singleton: 1200 (271) multiples: 1311 (316)	12–18 mo CA	370	Clinical/neurological, visual and hearing examination, BSID-II	7
Luo 2013 [62]	Cohort study	China	Upper middle	Not reported	Premature infants admitted to NICU	2008–2010	27–34	850–2300 g	12 mo CA	147	Clinical/neurological examination, PDMS-2	6
Burger 2011 [63]	Prospective cohort	South Africa	Upper middle	Jan–Dec 2004	Preterm infants (weighing <1250 g) and admitted to either the Level 2 neonatal wards, or to the neonatal intensive care unit	2004	30.0 (2.1)	1039.3 (160.5)	12 mo CA	115	Clinical/neurological examination, PDMS-2, AIMS	7
Rodriguez 2011 [64]	Cohort follow up	Brazil	Upper middle	2005–2006	Birth cohort undertaken in São Luís (1997/1998) and a follow-up survey conducted in 2005/2006	1997–1998	<37 weeks	Not available	7–9 y	190	SDQ	8
Gocer 2011 [65]	Cross-sectional	Turkey	Upper middle	Not available	Premature infants born with a birth weight of ≤1500 g and a gestational age ≤32 weeks	2002	≤32 weeks	≤1500 g	35.8 ± 2.3 mo	117	Clinical/neurological examination, DDST-II	6
Yang 2010 [66]	Follow up study	China	Upper middle	2000	Rural children aged 3–6 y	1993–1996	<37 weeks	Not available	3–6 y	4842	Anthropometry	6
deMoura 2010 [67]	Cohort follow up	Brazil	Upper middle	2006	All livebirths from mothers living in the urban area of Pelotas and in the Jardim America neighbourhood and were followed up at ages 3, 12 and 24 mo.	2004	<37 weeks	Not available	24 mo	532	BSDI	7
Mello 2009 [68]	Prospective cohort	Brazil	Upper middle	Not available	Premature newborns with birth weight <1500 g	2004–2006	29 week 6 d (2.0)	1126 (240)	12 mo CA	100	Clinical/neurological examination, BSID-II	7
Were 2006 [69]	Longitudinal Descriptive survey	Kenya	Lower middle	Not available	Infants born weighing 1000 g and 1500 g followed up until the age of 24 mo	2002	32.5 (2.4); range 28–36	1420 (93)	24 mo CA	120	Clinical/neurological examination, Dorothy Egan’s Model	8
Li 2001 [70]	Cross-sectional	China	Upper middle	May 1997–Dec 1998	Children aged 1–6 y from six provinces	1991–1997	<37	Not available	1–6 y	8256	Clinical/neurological examination	6
Airede 1992 [71]	Prospective study	Nigeria	Lower middle	1988	Preterm infants with ≤35 weeks GA and birth weight ≤1500 g	Not available	31 (1.3)	1125 (140)	2 y CA	159	Neurological, hearing and visual examination, GMDS	7

AIMS – Alberta Infant Motor Scale, ASQ – Ages & Stages Questionnaires-Third Edition, BSID-II – Bayley Scales of Infants Development II, BSID III – Bayley Scales of Infant and Toddler development, third edition, BSID – Battelle Screening Developmental Inventory, CPRS – Conners’ Parent Rating Scale-Revised, DASII – Developmental Assessment Scale for Indian Infants, DAWBA – Development and Well-Being Assessment, DCCC – Dyslexia Checklist for Chinese Children, DDST – Denver Development Screening Test, GARS-2 – Gilliam Autism Rating Scale, GDDS – Gesell Development Diagnosis Scale, GDS – Gesell Development Scale, GMDS – Griffiths Mental Development Scales, LDCDQ – Little Developmental Coordination Questionnaire, MABC-2 – Movement Assessment Battery for Children-second edition, M-CHAT – Modified Checklist for Autism in Toddlers, MD – median, NDSC – Neuropsychological Development Scale for Children, PDMS-2 – Peabody Developmental Motor Scale – 2, PRS – Pupil Rating Scale Revised, SDQ – Strengths and Difficulties Questionnaire, SIS – Sensory Integration Schedule, S-S – Sign-Significate relations; TDSC – Trivandrum Development Screening Chart, TDE – Teste de Desempenho Escolar, TT – Token Test, VABS – Vineland Adaptive Behavior Scales, x̄ – mean

### Primary outcomes

#### Neurodevelopmental impairment (NDI)

Eleven studies in five countries (China, India, Nigeria, Turkey and Vietnam) [27,31,35,40,41,48,52,54,55,61,71] reported on the prevalence of NDI in preterm-born children assessed at ages 12 to 24 months corrected age. The estimated pooled prevalence of NDI was 16% (95% CI = 11–21%, 11 studies, 3688 children, *I*^2^ = 93.91%, *P* = 0.00) (Figure S1 in the **Online Supplementary Document**).

#### Cerebral palsy (any severity)

Sixteen studies in six countries (China, India, Kenya, South Africa, Turkey and Vietnam) [27,30,31,35,40,41,47–49,52,61–63,65,69,70] reported prevalence of cerebral palsy in preterm-born children assessed at ages 12 months to six years. The estimated pooled prevalence was 5% (95% CI = 3–6, 15 studies, 12 148 children, *I*^2^ = 87.20%, *P* = 0.00) (Figure S2 in the **Online Supplementary Document**). A separate study in children with leukomalacia found a higher prevalence, 75% (95% CI = 67–83%) [30].

#### Hearing impairment/deafness

Seven studies [27,30,31,35,40,54,61] reported prevalence of hearing impairment/deafness, assessed at 12 months and four years. The estimated pooled prevalence of hearing impairment/deafness was 1% (95% CI = 0–2%, 7 studies, 3042 children, *I*^2^ = 75.82%, *P* = 0.00) (Figure S3 in the **Online Supplementary Document**).

#### Visual impairment/blindness

Eight studies [27,30,31,35,40,54,61,65] reported prevalence of visual impairment/blindness, assessing children from 12 months to four years – the pooled prevalence was 1% (95% CI = 0–2%, 8 studies, 3165 children, *I*^2^ = 74.47%, *P* = 0.00) (Figure S4 in the **Online Supplementary Document**).

#### Motor impairments

Two studies [25,43] in China reported on motor impairments from ages three to 10 years. The estimated pooled prevalence of motor impairment (developmental coordination disorder) was 13% (95% CI = 9–16%, 2 studies, 352 children, *I*^2^ = 0.00%, *P* = 0.35) (Figure S5 in the **Online Supplementary Document****)**. A further two studies in China screened children for suspected motor impairment using the Little Developmental Coordination Disorder Questionnaire (LDCDQ) with prevalences of 9 and 18% [32,38].

#### Developmental delays

Twenty-five studies reported on developmental delays across different domains [26,27,29–31,34–37,41,44,45,47–49,52,56,58,60–62,65,67–69]. The pooled prevalence of motor delays assessed between 12 months and 12 years was 13% (95% CI = 10–18%, 18 studies, 26 691 children, *I*^2^ = 98.67%, *P* = 0.00). The prevalence of overall motor delays was higher (19%, 95% CI = 13–26%) than gross motor delay (9%; 95% CI = 5–15%) and fine motor delay (8%; 95% CI = 5–12%) (Figure S6 in the **Online Supplementary Document****)**. For cognitive delays (any severity) assessed between 12 to 24 months’ corrected age, prevalence was 12% (95% CI = 8–26%, 8 studies, 2278 children, *I*^2^ = 97.21%, *P* = 0.00) (Figure S7 in the **Online Supplementary Document****)**, while for moderate-to-severe cognitive delays it was 9% (95% CI = 5–14%, 6 studies, 1964 children, *I*^2^ = 89.90%, *P* = 0.00). The pooled prevalence of language delay at 12 to 36 months was 12% (95% CI = 6–9%, 5 studies, 697 children, *I*^2^ = 86.55%, *P* = 0.00) (Figure S8 in the **Online Supplementary Document****)**. Global developmental delay at 12 to 24 months was 8% (95% CI = 4–14%, 4 studies, 953 children, *I*^2^ = 86.93%, *P* = 0.00) (Figure S9 in the **Online Supplementary Document**).

Some studies further used developmental scales with an overall general developmental score, such as the Grifﬁth’s Mental developmental scales, Brunet-Lézine scale and Battelle Screening Developmental Inventory [30,34,45,67]. Prevalence of developmental delays based on those scales ranged from 7.7 to 36%.

#### Behavioural and social-emotional disorders

Eight studies reported on a range of behavioural and emotional disorders. Two reported attention deficit/hyperactivity disorder (ADHD) [33,39] – the pooled prevalence was 4% (95% CI = 1–9%, 2 studies, 1527 children; *I*^2^ = 93.03%, *P* = 0.00) in children aged six to 12 years (Figure S10 in the **Online Supplementary Document**). Hyperactivity symptoms of ADHD using parent-rated Conners’ Hyperactivity Index were used in two studies, finding 4 and 12% [51,57]. Two studies screened for high risk of Autism Spectrum Disorder (ASD) using the Modified Checklist for Autism (M-CHAT) and the Gilliam Autism Rating scale (GARS-2) – prevalences ranged from 8.8 to 10% [28,47]. One study in Brazil estimated externalising and internalising disorders in children aged six and 11 years using the Development and Well-Being Assessment (DAWBA) tool, with a prevalence from 4 to 9% [50]. Another study in Brazil assessed behavioural problems (externalising and internalising problems) in children aged 7–9 years using the Strengths and Difﬁculties Questionnaire, finding a prevalence of 51% [64].

#### Learning difficulties

Two studies reported outcomes related to learning difficulties in preterm-born children. One from China estimated dyslexia in children from grade three to six of schooling and reported a prevalence of 4.2% [53]. The other, from Brazil, estimated academic performance in children ages eight to 10 years and the reported prevalence of poorer academic performance was about 32% [58].

### Secondary outcomes

#### Growth outcomes

Five studies in three countries (China, India and Vietnam) assessed growth outcomes in children at ages between 12 months and 15 years [31,42,46,59,66]. The pooled prevalence of underweight was 8% (95% CI = 1–21%, 9834 children, 5 studies, *I*^2^ = 98.71%, *P* = 0.01), while stunting was 9% (95% CI = 3–19%, 9834 children, 5 studies, *I*^2^ = 97.68%, *P* = 0.00) (Figure S11–12 in the **Online Supplementary Document****)**. Of note, one study [31] reported a very high prevalence of underweight (39%). After removing this study from the pooled analysis, the estimated prevalence decreased to 3% (95% CI = 2–5%).

### Additional analyses

Table S1–3 in the **Online Supplementary Document** summarises subgroup analyses, with stratifications based on mean gestational age, mean birthweight, and country income level. Results suggest the adverse outcomes increases as mean gestational age and birth weight decreases. Though this was not statistically significant for most outcomes. Higher prevalences were observed in upper-middle-income countries for some neurodevelopmental outcomes, but these results were not statistically significant. A ‘leave one out’ sensitivity analysis identified one outlier study where all children had leukomalacia in the neonatal period [30]. The prevalence of two outcomes (and their heterogeneity) decreased by excluding it.

We conducted additional sensitivity analyses, excluding studies of moderate quality. This yielded results within or close to the confidence interval of the overall prevalence estimates, with minimal change in the *I*^2^ statistic (Table S4 in the **Online Supplementary Document****)**.

Funnel plots for the pooled estimates (Figure S13 in the **Online Supplementary Document**) and Egger’s test suggests that publication bias might be present for the cerebral palsy outcome.

## DISCUSSION

In this systematic review and meta-analysis, we sought to estimate the prevalence of adverse neurodevelopmental outcomes in children after preterm birth in LMICs. We found data for 72 974 preterm-born children from 12 mostly upper-middle-income countries. The pooled prevalence estimates of neurodevelopmental outcomes for these children varied from 1 to 16% – the highest prevalence estimates were for overall NDI and cognitive developmental delays (16%) while visual and hearing impairments were lowest (1%).

We estimated the prevalence of NDI to be 16% – this is lower than the 29% reported by Ramaswamy et al in 2021 using four studies [16], and the median prevalence of 21.4% reported by Milner et al in 2015 [17] based on 16 studies. The difference is likely attributable to population differences – with the first review focusing on extremely preterm infants only, while the second included preterm and/or very low birth weight children only. We found the prevalence of cerebral palsy in preterm-born children in LMICs was 5%. A previous meta-analysis reported that 6.8% of children born <32 weeks’ gestation and/or born very low birth weight had cerebral palsy, though this varied by setting – 4.9% in upper-middle-income countries and 9.5% in lower-middle-income countries [72]. We found a somewhat lower prevalence of hearing and visual impairment than that of other reported outcomes and similar findings were also observed in other international studies [73,74]. However, it is worth noting that these deficits can further negatively impact children's cognitive, academic, and socio-emotional development in the future. We estimate that childhood developmental delays across different domains range from 8 to 13%, including large differences in the prevalences between the studies based on developmental measurement tools. A 2018 meta-analysis, composed primarily of studies from high-income countries, found higher prevalence rates of cognitive and motor delays (16.9 and 20.6%, respectively) in very preterm and/or low birth weight children [72]. Although our estimates were lower than those in previous studies, likely due to differences in preterm populations and study settings – they remain substantially higher than those in the general population. This is consistent with findings from a review by Bitta et al. reporting neurodevelopmental disorders in LMICs [75].

A growing body of evidence suggests that preterm birth is associated with an increased risk of behavioural problems, particularly attention deficits, social-emotional difficulties and internalising problems [76]. These outcomes represent crucial long-term concerns that can significantly impact quality of life and overall development. However, we found few studies reporting behavioural and emotional outcomes. For those that did report it, prevalences varied across studies, and most used generic behavioural or emotional screening tools, rather than a comprehensive clinical diagnostic approach. Future research incorporating both screening and diagnostic assessment is needed to better understand the prevalence and magnitude of these outcomes in LMICs.

Across all outcomes there was substantive between-study heterogeneity, which could not be fully explained by subgroup analyses stratified by mean gestational age, mean birth weight, and country income level, or by sensitivity analyses excluding lower-quality studies. This could be due to other factors such as maternal and neonatal comorbidities, study setting, intensity of perinatal care, age at assessment, outcome measurement tools or tool thresholds. Overall, the majority ( ~ 60%) of the included studies were assessed as having high methodological quality, and importantly, none were rated as low quality, which enhances the reliability of our pooled estimates. Yet, it is essential to consider potential biases that might arise from variations in the quality appraisal scores of the studies.

The occurrence of neurodevelopmental outcomes is shaped by a complex interplay between sociodemographic, economic, environmental, and health system factors, particularly in LMICs [77]. These multidimensional determinants vary across settings, likely contributing to differences in prevalence and severity of adverse neurodevelopmental outcomes in children. For instance, disparities in health care infrastructure, access to early intervention services, and socioeconomic conditions would influence these outcomes. Additionally, sociocultural norms and cultural differences can further complicate the accurate identification and reporting of neurodevelopmental conditions. Parental perceptions and expectations regarding developmental milestones and behaviours are often influenced by cultural and societal beliefs and norms, affecting the recognition and reporting of developmental concerns [78]. This is further compounded by the widespread stigma associated with neurodevelopmental disabilities in many settings, leading to potential underreporting. This highlights the importance of considering these factors while interpreting the reported prevalence estimates.

We observed a wide variability in the use of measurement tools and cut-off thresholds across the included studies. Lack of standardised, validated tools for childhood developmental assessments has long been a major challenge for LMICs [79]. While attempts have been made to introduced standardised tools from high-income settings into LMIC contexts, this has been limited by cultural and linguistic differences, differences in score interpretation and lack of local adaptation and validation. Hence, future studies should emphasise developing or adapting neurodevelopmental assessment tools that are appropriate to local cultural norms, and conducting validation studies to ensure their accuracy in diverse settings.

Additionally, data from the included studies are mostly limited to assessment during early childhood. Since many of the impairments can persist throughout the life course, continuous monitoring and further longitudinal studies are needed to track these outcomes into adolescence and adulthood. Such research would offer a more comprehensive understanding of the long-term impacts, help identify critical windows for intervention, and inform strategies to improve support and outcomes in these children over time.

To the best of our knowledge, out study provides the most up-to-date and comprehensive estimate of a broad spectrum of adverse neurodevelopmental outcomes in preterm born children in LMICs. Our systematic review employed a rigorous and comprehensive search strategy with no language restrictions across six databases. Additionally, by setting a minimum sample size threshold of 100 children, we ensured the exclusion of less representative studies and enhancing the robustness of our findings. However, several limitations need to considered while interpreting our results. First, we observed substantial heterogeneity across studies, particularly in the assessment of neurodevelopmental outcomes. The included studies employed a wide range of measurement tools, cut-off thresholds, and diagnostic criteria, which often complicates direct comparisons and may introduce variability in the prevalence estimates. Therefore, these estimates should be interpreted with caution, considering the underlying variability. Second, we identified studies from only 12 countries, mostly upper-middle-income countries. Limited representation from low-and lower-middle-income countries indicates geographical bias in the evidence base, which could potentially impact generalisability of the overall findings to such countries. Health data availability remains a critical challenge for many LMICs. The lack of comprehensive and high-quality data from these countries limits our understanding of the burden of adverse neurodevelopmental outcomes in childhood. Given the burden of preterm birth in these countries is substantial, it is plausible that our prevalence estimates might underrepresent the true burden, particularly for low-income countries. Data from low-and lower-middle income countries are needed, as are studies on under-explored outcomes, such as behavioural and emotional disorders. Third, the observed publication bias in cerebral palsy underscores the possibility of selective publication for positive findings. This bias can lead to an imprecise pooled estimation for this outcome.

Overall, findings from our study can guide governments, policymakers, public health researchers and other stakeholders in optimising resource allocation, address service gaps and planning for targeted activities for preterm-born children with adverse neurodevelopmental outcomes in LMICs. Strong advocacy programmes and policy-level initiatives are vital to raise awareness on the magnitude of this problem and establishing platforms to ensure timely intervention and rehabilitation services that are affordable, accessible, and inclusive. Prioritising routine screening and surveillance strategies is essential to facilitate early detection of the adverse outcomes and improve long-term developmental trajectories in these children. Additionally, standardised tools and guidelines along with appropriate training of health care providers are needed for early detection, referral and treatment as well as ensuring quality of care.

## CONCLUSION

In this systematic review and meta-analysis, our results underscore the substantial burden of long-term adverse neurodevelopmental outcomes in children after preterm birth in LMICs. These findings highlight the critical need for early diagnosis, timely intervention, follow up and rehabilitation to mitigate these adverse outcomes. Furthermore, our findings can inform targeted public health and clinical strategies to meet the needs in these settings. Future research should prioritise low-and lower-middle-income countries, focus on longer-term and comprehensive neurodevelopmental assessment studies, and ensure that measurement tools are culturally appropriate, standardised and validated.

## Additional material


Online Supplementary Document
