# Supplementary material for: The prevalence of long-term neurodevelopmental outcomes in preterm-born children in low- and middle-income countries: a systematic review and meta-analysis of developmental outcomes in 72 974 preterm-born children
Source: J Glob Health. 2025 Apr 4;15:04106. doi: 10.7189/jogh.15.04106 (PMC11969289; doi:10.7189/jogh.15.04106)
Supplement: Online Supplementary Document [file jogh-15-04106-s001.pdf]

Appendix S1: Search Strategy

Ovid MEDLINE(R)	Embase Classic+Embase	APA PsycInfo
1 obstetric labor, premature/ or premature birth/<br>2 infant, premature/ or infant, extremely premature/<br>3 ((preterm or pre-term or prematur* or pre-matur*) adj2 (infant* or birth* or child* or deliver* or labour or labor or born or baby or babies or neonate* or newborn*)).mp.<br>4 (((" 24" or "25" or "26" or "27" or "28" or "29" or "30" or "31" or "32" or "33" or "34" or "35" or "36") adj weeks) and (gestation* or pregnan*)).mp.<br>5 1 or 2 or 3 or 4<br>6 Neurodevelopmental Disorders/<br>7 Developmental Disabilities/ or Cerebral Palsy/ or Intellectual Disability/ or hearing disorders/ or hearing loss/ or deafness/ or vision disorders/ or blindness/ or Learning Disabilities/ or Intelligence/ or Academic Performance/ or Psychomotor Performance/ or Psychomotor Disorders/ or Cognition Disorders/ or Motor Skills Disorders/<br>8 adolescent development/ or child development/<br>9 nervous system diseases/ or central nervous system diseases/ or attention deficit disorder with hyperactivity/ or child behavior disorders/ or child development disorders, pervasive/ or autism spectrum disorder/ or autistic disorder/<br>10 ((longterm or long-term or development* or neurodevelopmental or intellectual or vision or hearing or psychomotor or cognitive or motor or mental or attention or academic or learning) adj3 (effect* or outcome* or disorder* or disabilit* or impairment*)).mp.<br>11 (cerebral palsy or blindness or deafness or autistic trait* or ADHD or autism).mp.<br>12 (academic adj (performance or achievement* or success)).mp.	1 premature labor/ or "immature and premature labor"/<br>2 prematurity/<br>3 ((preterm or pre-term or prematur* or pre-matur*) adj2 (infant* or birth* or child* or deliver* or labour or labor or born or baby or babies or neonate* or newborn*)).mp.<br>4 ((("24" or "25" or "26" or "27" or "28" or "29" or "30" or "31" or "32" or "33" or "34" or "35" or "36") adj weeks) and (gestation* or pregnan*)).mp.<br>5 1 or 2 or 3 or 4<br>6 developmental disorder/ or developmental delay/<br>7 cerebral palsy/ or mental deficiency/ or intellectual impairment/ or hearing impairment/ or hearing disorder/ or unilateral hearing loss/ or functional hearing loss/ or perception deafness/ or visual disorder/ or blindness/ or deafblindness/ or partial blindness/ or learning disorder/ or intelligence/ or academic achievement/ or psychomotor performance/ or psychomotor activity/ or psychomotor disorder/ or cognitive defect/ or motor dysfuntion/ or motor development/<br>8 adolescent development/ or child development/<br>9 central nervous system disease/ or neurologic disease/ or attention deficit hyperactivity disorder/ or behavior disorder/ or autism/<br><br>10 ((longterm or long-term or development* or neurodevelopmental or intellectual or vision or hearing or psychomotor or cognitive or motor or mental or attention or academic or	1 Premature Birth/<br>2 ((preterm or pre-term or prematur* or pre-matur*) adj2 (infant* or birth* or child* or deliver* or labour or labor or born or baby or babies or neonate* or newborn*)).mp.<br>3 ((("24" or "25" or "26" or "27" or "28" or "29" or "30" or "31" or "32" or "33" or "34" or "35" or "36") adj weeks) and (gestation* or pregnan*)).mp.<br>4 1 or 2 or 3<br>5 Neurodevelopmental Disorders/<br>6 developmental disabilities/ or delayed development/ or intellectual development disorder/ or specific language impairment/ or cerebral palsy/ or deaf/ or hearing disorders/ or blind/ or vision disorders/ or learning disabilities/ or learning disorders/ or intelligence/ or intellectual development/ or academic achievement/ or cognitive ability/ or cognitive impairment/ or cognitive development/ or psychomotor development/ or motor development/<br>7 early childhood development/ or childhood development/ or adolescent development/<br>8 central nervous system disorders/ or nervous system disorders/ or autism spectrum disorders/ or autistic traits/ or attention deficit disorder with hyperactivity/ or attention deficit disorder/<br>9 ((longterm or long-term or development* or neurodevelopmental or intellectual or vision or hearing or psychomotor or cognitive or motor or mental or attention or academic or learning) adj3 (effect* or outcome* or disorder* or disabilit* or impairment*)).mp.<br>10 (cerebral palsy or blindness or deafness or autistic trait* or ADHD or autism).mp.<br>11 (academic adj (performance or achievement* or success)).mp.

13 (education* adj (performance or achievement* or success)).mp.<br>14 ((child* or adolescent) adj development).mp.<br>15 6 or 7 or 8 or 9 or 10 or 11 or 12 or 13 or 14<br>16 Developing Countries/<br>17 (Afghanistan* or Albania* or Algeria* or Angola* or Argentina* or Armenia* or Azerbaijan* or Bangladesh* or Belarus* or Beliz* or Benin* or Bhutan* or Bolivia* or Bosnia* or Herzegovin* or Botswan* or Brazil* or Bulgaria* or Burkina* or Burundi* or Cabo Verde* or Cape Verde* or Cambodia* or Cameroon* or Central African or Chad* or China or Chinese or Colombia* or Comor* or Congo* or Costa Rica* or Cote d'Ivoir* or Ivory Coast or Cuba* or Djibouti* or Dominica* or Ecuador* or Egypt* or El Salvador* or Eritrea* or Ethiopia* or Fiji* or Gabon* or Gambia* or Georgia* or Ghana* or Grenad* or Guatemala* or Guinea* or Guyan* or Haiti* or Hondura* or India* or Indonesia* or Iran* or Iraq* or Jamaica* or Jordan* or Kazakhstan* or Kenya* or Kiribati* or Korea* or Kosov* or Kyrgyz* or Lao* or Leban* or Lesotho* or Liberia* or Libya* or Macedonia* or Madagascar* or Malawi* or Malaysia* or Maldiv* or Mali* or Marshall Island* or Mauritania* or Mauriti* or Mexic* or Micronesia* or Moldova* or Mongolia* or Montenegr* or Morocc* or Mozambi* or Myanma* or Burmese or Namibia* or Nepal* or Nicaragua* or Niger* or Pakistan* or Palau* or Papua New Guinea* or Paraguay* or Peru* or Philippines or Filipino or Romania* or Rwanda* or Samoa* or Sao Tome* or Senegal* or Serbia* or Sierra Leon* or Solomon Island* or Somalia* or South Africa* or Sudan* or Sri Lanka* or St Lucia* or Saint Lucia or St Vincent or Saint Vincent or Grenadines or Surinam* or Swazi* or Syria* or Tajikistan* or Tanzania* or Thai* or Timor* or Togo* or Tonga* or Tunisia* or Turk* or Tuvalu* or Uganda* or Ukrain* or Uzbekistan* or Vanuatu* or Venezuela* or	learning) adj3 (effect* or outcome* or disorder* or disabilit* or impairment*)).mp.<br>11 (cerebral palsy or blindness or deafness or autistic trait* or ADHD or autism).mp.<br>12 (academic adj (performance or achievement* or success)).mp.<br>13 (education* adj (performance or achievement* or success)).mp.<br>14 ((child* or adolescent) adj development).mp.<br>15 6 or 7 or 8 or 9 or 10 or 11 or 12 or 13 or 14<br>16 developing country/<br><br>17 (Afghanistan* or Albania* or Algeria* or Angola* or Argentina* or Armenia* or Azerbaijan* or Bangladesh* or Belarus* or Beliz* or Benin* or Bhutan* or Bolivia* or Bosnia* or Herzegovin* or Botswan* or Brazil* or Bulgaria* or Burkina* or Burundi* or Cabo Verde* or Cape Verde* or Cambodia* or Cameroon* or Central African or Chad* or China or Chinese or Colombia* or Comor* or Congo* or Costa Rica* or Cote d'Ivoir* or Ivory Coast or Cuba* or Djibouti* or Dominica* or Ecuador* or Egypt* or El Salvador* or Eritrea* or Ethiopia* or Fiji* or Gabon* or Gambia* or Georgia* or Ghana* or Grenad* or Guatemala* or Guinea* or Guyan* or Haiti* or Hondura* or India* or Indonesia* or Iran* or Iraq* or Jamaica* or Jordan* or Kazakhstan* or Kenya* or Kiribati* or Korea* or Kosov* or Kyrgyz* or Lao* or Leban* or Lesotho* or Liberia* or Libya* or Macedonia* or Madagascar* or Malawi* or Malaysia* or Maldiv* or Mali* or Marshall Island* or Mauritania* or Mauriti* or Mexic* or Micronesia* or Moldova* or Mongolia* or	12 (education* adj (performance or achievement* or success)).mp.<br>13 ((child* or adolescent) adj development).mp.<br>14 5 or 6 or 7 or 8 or 9 or 10 or 11 or 12 or 13<br>15 Developing Countries/<br>16 (Afghanistan* or Albania* or Algeria* or Angola* or Argentina* or Armenia* or Azerbaijan* or Bangladesh* or Belarus* or Beliz* or Benin* or Bhutan* or Bolivia* or Bosnia* or Herzegovin* or Botswan* or Brazil* or Bulgaria* or Burkina* or Burundi* or Cabo Verde* or Cape Verde* or Cambodia* or Cameroon* or Central African or Chad* or China or Chinese or Colombia* or Comor* or Congo* or Costa Rica* or Cote d'Ivoir* or Ivory Coast or Cuba* or Djibouti* or Dominica* or Ecuador* or Egypt* or El Salvador* or Eritrea* or Ethiopia* or Fiji* or Gabon* or Gambia* or Georgia* or Ghana* or Grenad* or Guatemala* or Guinea* or Guyan* or Haiti* or Hondura* or India* or Indonesia* or Iran* or Iraq* or Jamaica* or Jordan* or Kazakhstan* or Kenya* or Kiribati* or Korea* or Kosov* or Kyrgyz* or Lao* or Leban* or Lesotho* or Liberia* or Libya* or Macedonia* or Madagascar* or Malawi* or Malaysia* or Maldiv* or Mali* or Marshall Island* or Mauritania* or Mauriti* or Mexic* or Micronesia* or Moldova* or Mongolia* or Montenegr* or Morocc* or Mozambi* or Myanma* or Burmese or Namibia* or Nepal* or Nicaragua* or Niger* or Pakistan* or Palau* or Papua New Guinea* or Paraguay* or Peru* or Philippines or Filipino or Romania* or Rwanda* or Samoa* or Sao Tome* or Senegal* or Serbia* or Sierra Leon* or Solomon Island* or Somalia* or South Africa* or Sudan* or Sri Lanka* or St Lucia* or Saint Lucia or St Vincent or Saint Vincent or Grenadines or Surinam* or Swazi* or Syria* or Tajikistan* or Tanzania* or Thai* or Timor* or Togo* or Tonga* or Tunisia* or Turk* or Tuvalu* or Uganda* or Ukrain* or Uzbekistan* or Vanuatu* or Venezuela* or

Vietnam* or viet-nam* or West Bank or Gaza or Yemen* or Zambia* or Zimbabwe* or Russia* or Yugoslavia* or USSR or Soviet* or Byelarus*).mp.<br>18 (africa* or asia* or caribbean or central america* or latin america* or south america* or melanesia* or micronesia* or polynesia*).mp.<br>19 (resource-limit* or resource-poor or low-resource* or limited-resource* or resource-constrain* or constrain*-resource* or under-resource* or poor*-resource* or resource-scarce* or scarce*-resource* or low-income or middle-income or lowincome or middleincome or (low* adj3 middle-income)).mp.<br>20 (((developing or underdeveloped or under-developed or emerging or less-developed or least-developed or less-economically developed or least-economically developed or less-affluent or least-affluent or deprived or poor) adj (country or countries or nation? or region? or economy or economies)) or ((developing or underdeveloped or under-developed or less-developed or least-developed) adj (population* or world)) or (third-world* or thirdworld* or 3rd-world* or lmic or lmics or lami countr* or lalmi countr* or transitional countr*) or (low* adj (gdp or gnp or gross domestic or gross national)) or ((underserved or under-served) adj (countr* or nation? or population*))).mp.<br>21 16 or 17 or 18 or 19 or20<br>22 5 and 15 and 21	Montenegr* or Morocc* or Mozambi* or Myanma* or Burmese or Namibia* or Nepal* or Nicaragua* or Niger* or Pakistan* or Palau* or Papua New Guinea* or Paraguay* or Peru* or Philippines or Filipino or Romania* or Rwanda* or Samoa* or Sao Tome* or Senegal* or Serbia* or Sierra Leon* or Solomon Island* or Somalia* or South Africa* or Sudan* or Sri Lanka* or St Lucia* or Saint Lucia or St Vincent or Saint Vincent or Grenadines or Surinam* or Swazi* or Syria* or Tajikistan* or Tanzania* or Thai* or Timor* or Togo* or Tonga* or Tunisia* or Turk* or Tuvalu* or Uganda* or Ukrain* or Uzbekistan* or Vanuatu* or Venezuela* or Vietnam* or viet-nam* or West Bank or Gaza or Yemen* or Zambia* or Zimbabwe* or Russia* or Yugoslavia* or USSR or Soviet* or Byelarus*).mp.<br>18 (africa* or asia* or caribbean or central america* or latin america* or south america* or melanesia* or micronesia* or polynesia*).mp.<br>19 (resource-limit* or resource-poor or low-resource* or limited-resource* or resource-constrain* or constrain*-resource* or under-resource* or poor*-resource* or resource-scarce* or scarce*-resource* or low-income or middle-income or lowincome or middleincome or (low* adj3 middle-income)).mp.<br>20 (((developing or underdeveloped or under-developed or emerging or less-developed or least-developed or less-economically developed or least-economically developed or less-affluent or least-affluent or deprived or poor) adj (country or countries or nation? or region? or economy or economies)) or	Vietnam* or viet-nam* or West Bank or Gaza or Yemen* or Zambia* or Zimbabwe* or Russia* or Yugoslavia* or USSR or Soviet* or Byelarus*).mp. 488938<br>17 (africa* or asia* or caribbean or central america* or latin america* or south america* or melanesia* or micronesia* or polynesia*).mp.<br>18 (resource-limit* or resource-poor or low-resource* or limited-resource* or resource-constrain* or constrain*-resource* or under-resource* or poor*-resource* or resource-scarce* or scarce*-resource* or low-income or middle-income or lowincome or middleincome or (low* adj3 middle-income)).mp.<br>19 (((developing or underdeveloped or under-developed or emerging or less-developed or least-developed or less-economically developed or least-economically developed or less-affluent or least-affluent or deprived or poor) adj (country or countries or nation? or region? or economy or economies)) or ((developing or underdeveloped or under-developed or less-developed or least-developed) adj (population* or world)) or (third-world* or thirdworld* or 3rd-world* or lmic or lmics or lami countr* or lalmi countr* or transitional countr*) or (low* adj (gdp or gnp or gross domestic or gross national)) or ((underserved or under-served) adj (countr* or nation? or population*))).mp.<br>20 15 or 16 or 17 or 18 or 19<br>21 4 and 14 and 20

	((developing or underdeveloped or under-developed or less-developed or least-developed) adj (population* or world)) or (third-world* or thirdworld* or 3rd-world* or lmic or lmics or lami countr* or lalmi countr* or transitional countr*) or (low* adj (gdp or gnp or gross domestic or gross national)) or ((underserved or under-served) adj (countr* or nation? or population*))).m<br>21 16 or 17 or 18 or 19 or 20<br>22 5 and 15 and 21	
**CINAHL plus**	**Web of Science**	**Scopus**
S1 (MH "Childbirth, Premature") OR (MH "Labor, Premature")<br>S2 (MH "Infant, Premature")<br>S3 (preterm or "pre-term" or prematur* or "pre-matur*") N2 (infant* or birth* or child* or deliver* or labour or labor or born or baby or babies or neonate* or newborn*)<br>S4 ((("24" or "25" or "26" or "27" or "28" or "29" or "30" or "31" or "32" or "33" or "34" or "35" or "36") N0 weeks) and (gestation* or pregnan*))<br>S5 S1 OR S2 OR S3 OR S4<br>S6 (MH "Developmental Disabilities") OR (MH "Intellectual Disability") OR (MH "Learning Disorders") OR (MH "Motor Skills") OR (MH "Motor Skills Disorders") OR (MH "Child Development Disorders, Pervasive") OR (MH "Mental Disorders Diagnosed in Childhood") OR (MH "Social Behavior Disorders") OR (MH "Child Behavior Disorders") OR (MH "Hearing Disorders") OR (MH "Deaf-Blind Disorders") OR (MH "Hearing Loss, Partial") OR (MH "Hearing Loss, Sensorineural") OR (MH "Hearing Loss, Central") OR (MH "Hearing Loss, Conductive") OR (MH "Vision Disorders") OR (MH "Intelligence") OR (MH "Academic Performance") OR (MH "Psychomotor Performance") OR (MH "Psychomotor Disorders") OR (MH "Cognition Disorders")<br>S7 (MH "Central Nervous System Diseases") OR (MH	1 TS=((preterm or pre-term or prematur* or pre-matur*) NEAR/2 (infant* or birth* or child* or deliver* or labour or labor or born or baby or babies or neonate* or newborn*))<br>2 TS=(((("24" or "25" or "26" or "27" or "28" or "29" or "30" or "31" or "32" or "33" or "34" or "35" or "36") NEAR/0 weeks) and (gestation* or pregnan*)))<br>3 #1 OR #2<br>4 TS=((longterm or long-term or development* or neurodevelopmental or intellectual or vision or hearing or psychomotor or cognitive or motor or mental or attention or academic or learning) NEAR/2 (effect* or outcome* or disorder* or disabilit* or impairment*)) 5 TS=("cerebral palsy" or blindness or deafness or "autistic trait*" or ADHD or autism) 242342<br>6 TS=(academic NEAR/0 (performance or achievement* or success))<br>7 TS=(education* NEAR/0 (performance or achievement* or success))<br>8 TS=((child* or adolescent) NEAR/0 development)<br>9 #4 OR #5 OR #6 OR #7 OR #8<br>10 TS=(Afghanistan* or Albania* or Algeria* or Angola* or Argentina* or	1 TITLE-ABS-KEY ((preterm OR pre-term OR prematur* OR pre-matur*) W/1 (infant* OR birth* OR child* OR deliver* OR labour OR labor OR born OR baby OR babies OR neonate* OR newborn*))<br>2 TITLE-ABS-KEY (("24" OR "25" OR "26" OR "27" OR "28" OR "29" OR "30" OR "31" OR "32" OR "33" OR "34" OR "35" OR "36") W/0 (weeks) AND (gestation* OR pregnan*))<br>3 1 OR 2<br>4 TITLE-ABS-KEY ((longterm OR “long-term” OR development* OR neurodevelopmental OR intellectual OR vision OR hearing OR psychomotor OR cognitive OR motor OR mental OR attention OR academic OR learning) W/2 (effect* OR outcome* OR disorder* OR disabilit* OR impairment*))<br>5 TITLE-ABS-KEY (“cerebral palsy" OR blindness OR deafness OR "autistic trait*" OR adhd OR autism)<br>6 TITLE-ABS-KEY (academic W/0 (performance OR achievement* OR succes))<br>7 TITLE-ABS-KEY (education* W/0 (performance OR achievement* OR success))<br>8 TITLE-ABS-KEY ((child* OR adolescent) W/0 development) 9 4 OR 5 OR 6 OR 7 OR 8<br>10 TITLE-ABS-KEY (afghanistan* OR albania* OR algeria* OR angola* OR argentina* OR armenia* OR azerbaijan* OR bangladesh* OR belarus* OR beliz* OR benin* OR bhutan* OR bolivia* OR bosnia* OR herzegovin* OR

"Nervous System Diseases") OR (MH "Autistic Disorder") OR (MH "Attention Deficit Hyperactivity Disorder")<br>S8 (MH "Child Development: Middle Childhood (6-11 Years) (Iowa NOC)") OR (MH "Child Development") OR (MH "Child Development: Adolescence (12-17 Years) (Iowa NOC)") OR (MH "Child Development: 5 Years (Iowa NOC)") OR (MH "Child Development: 6 Months (Iowa NOC)") OR (MH "Child Development: 4 Years (Iowa NOC)") OR (MH "Child Development: 3 Years (Iowa NOC)") OR (MH "Child Development: 2 Years (Iowa NOC)") OR (MH "Child Development: 12 Months (Iowa NOC)") OR (MH "Adolescent Development") 31,284<br>S9 (longterm or "long-term" or development* or neurodevelopmental or intellectual or vision or hearing or psychomotor or cognitive or motor or mental or attention or academic or learning) N3 (effect* or outcome* or disorder* or disabilit* or impairment*) 365,134<br>S10 "cerebral palsy" or blindness or deafness or "autistic trait*" or ADHD or autism<br>S11 (academic N0 (performance or achievement* or success))<br>S12 (education* N0 (performance or achievement* or success))<br>S13 (child* or adolescent) N0 development<br>S14 S6 OR S7 OR S8 OR S9 OR S10 OR S11 OR S12 OR S13<br>S15 (MH "Developing Countries") OR (MH "Low and Middle Income Countries")<br>S16 Afghanistan* OR Albania* OR Algeria* OR Angola* OR Argentina* OR Armenia* OR Azerbaijan* OR Bangladesh* OR Belarus* OR Beliz* OR Benin* OR Bhutan* OR Bolivia* OR Bosnia* OR Herzegovin* OR Botswan* OR Brazil* OR Bulgaria* OR Burkina* OR Burundi* OR Cabo Verde* OR Cape Verde* OR Cambodia* OR Cameroon* OR “Central African” OR Chad* OR China OR Chinese OR Colombia* OR Comor* OR	Armenia* or Azerbaijan* or Bangladesh* or Belarus* or Beliz* or Benin* or Bhutan* or Bolivia* or Bosnia* or Herzegovin* or Botswan* or Brazil* or Bulgaria* or Burkina* or Burundi* or “Cabo Verde*” or “Cape Verde*” or Cambodia* or Cameroon* or "Central African" or Chad* or China or Chinese or Colombia* or Comor* or Congo* or "Costa Rica*" or "Cote d'Ivoir*" or "Ivory Coast" or Cuba* or Djibouti* or Dominica* or Ecuador* or Egypt* or "El Salvador*" or Eritrea* or Ethiopia* or Fiji* or Gabon* or Gambia* or Georgia* or Ghana* or Grenad* or Guatemala* or Guinea* or Guyan* or Haiti* or Hondura* or India* or Indonesia* or Iran* or Iraq* or Jamaica* or Jordan* or Kazakhstan* or Kenya* or Kiribati* or Korea* or Kosov* or Kyrgyz* or Lao* or Leban* or Lesotho* or Liberia* or Libya* or Macedonia* or Madagascar* or Malawi* or Malaysia* or Maldiv* or Mali* or "Marshall Island*" or Mauritania* or Mauriti* or Mexic* or Micronesia* or Moldova* or Mongolia* or Montenegr* or Morocc* or Mozambi* or Myanma* or Burmese or Namibia* or Nepal* or Nicaragua* or Niger* or Pakistan* or Palau* or "Papua New Guinea*" or Paraguay* or Peru* or Philippines or Filipino or Romania* or Rwanda* or Samoa* or Sao Tome* or Senegal* or Serbia* or Sierra Leon* or "Solomon Island*" or Somalia* or "South Africa*" or Sudan* or "Sri Lanka*" or "St Lucia*" or "Saint Lucia" or "St Vincent" or "Saint Vincent" or Grenadines or Surinam* or Swazi* or Syria* or Tajikistan* or Tanzania* or Thai* or Timor* or Togo* or Tonga* or Tunisia* or Turk* or Tuvalu* or Uganda* or	botswan* OR brazil* OR bulgaria* OR burkina* OR burundi* OR “cabo verde*” OR “cape verde*” OR cambodia* OR cameroon* OR "central african" OR chad* OR china OR chinese OR colombia* OR comor* OR congo* OR "costa rica*" OR "cote d'ivoir*" OR "ivory coast" OR cuba* OR djibouti* OR dominica* OR ecuador* OR egypt* OR "el salvador*" OR eritrea* OR ethiopia* OR fiji* OR gabon* OR gambia* OR georgia* OR ghana* OR grenad* OR guatemala* OR guinea* OR guyan* OR haiti* OR hondura* OR india* OR indonesia* OR iran* OR iraq* OR jamaica* OR jordan* OR kazakhstan* OR kenya* OR kiribati* OR korea* OR kosov* OR kyrgyz* OR lao* OR leban* OR lesotho* OR liberia* OR libya* OR macedonia* OR madagascar* OR malawi* OR malaysia* OR maldiv* OR mali* OR "marshall island*" OR mauritania* OR mauriti* OR mexic* OR micronesia* OR moldova* OR mongolia* OR montenegr* OR morocc* OR mozambi* OR myanma* OR burmese OR namibia* OR nepal* OR nicaragua* OR niger* OR pakistan* OR palau* OR "papua new guinea*" OR paraguay* OR peru* OR philippines OR filipino OR romania* OR rwanda* OR samoa* OR "sao tome*" OR senegal* OR serbia* OR "sierra leon*" OR "solomon island*" OR somalia* OR "south africa*" OR sudan* OR "sri lanka*" OR "st lucia*" OR "saint lucia" OR "st vincent" OR "saint vincent" OR grenadines OR surinam* OR swazi* OR syria* OR tajikistan* OR tanzania* OR thai* OR timor* OR togo* OR tonga* OR tunisia* OR turk* OR tuvalu* OR uganda* OR ukrain* OR uzbekistan* OR vanuatu* OR venezuela* OR vietnam* OR "viet-nam*" OR "west bank" OR gaza OR yemen* OR zambia* OR zimbabwe* OR russia* OR yugoslavia* OR ussr OR soviet* OR byelarus*)<br>11 TITLE-ABS-KEY (africa* OR asia* OR caribbean OR "central america*" OR "latin america*" OR "south america*" OR melanesia* OR micronesia* OR polynesia*)

Congo* OR “Costa Rica*” OR “Cote d'Ivoir*” OR “Ivory Coast” OR Cuba* OR Djibouti* OR Dominica* OR Ecuador* OR Egypt* OR “El Salvador*” OR Eritrea* OR Ethiopia* OR Fiji* OR Gabon* OR Gambia* OR Georgia* OR Ghana* OR Grenad* OR Guatemala* OR Guinea* OR Guyan* OR Haiti* OR Hondura* OR India* OR Indonesia* OR Iran* OR Iraq* OR Jamaica* OR Jordan* OR Kazakhstan* OR Kenya* OR Kiribati* OR Korea* OR Kosov* OR Kyrgyz* OR Lao* OR Leban* OR Lesotho* OR Liberia* OR Libya* OR Macedonia* OR Madagascar* OR Malawi* OR Malaysia* OR Maldiv* OR Mali* OR “Marshall Island*” OR Mauritania* OR Mauriti* OR Mexic* OR Micronesia* OR Moldova* OR Mongolia* OR Montenegr* OR Morocc* OR Mozambi* OR Myanma* OR Burmese OR Namibia* OR Nepal* OR Nicaragua* OR Niger* OR Pakistan* OR Palau* OR “Papua New Guinea*” OR Paraguay* OR Peru* OR Philippines OR Filipino OR Romania* OR Rwanda* OR Samoa* OR “Sao Tome*” OR Senegal* OR Serbia* OR “Sierra Leon*” OR “Solomon Island*” OR Somalia* OR “South Africa*” OR Sudan* OR “Sri Lanka*” OR “St Lucia*” OR “Saint Lucia” OR “St Vincent” OR “Saint Vincent” OR Grenadines OR Surinam* OR Swazi* OR Syria* OR Tajikistan* OR Tanzania* OR Thai* OR Timor* OR Togo* OR Tonga* OR Tunisia* OR Turk* OR Tuvalu* OR Uganda* OR Ukrain* OR Uzbekistan* OR Vanuatu* OR Venezuela* OR Vietnam* OR “viet-nam*” OR “West Bank” OR Gaza OR Yemen* OR Zambia* OR Zimbabwe* OR Russia* OR Yugoslavia* OR USSR OR Soviet* OR Byelarus* 887,671<br>S17 africa* or asia* or caribbean or “central america*” or “latin america*” or “south america*” or melanesia* or micronesia* or polynesia*<br>S18 “resource-limit*” or “resource-poor” or “low-resource*” or “limited-resource*” or “resource-constrain*” or “constrain*-	Ukrain* or Uzbekistan* or Vanuatu* or Venezuela* or Vietnam* or "viet-nam*" or "West Bank" or Gaza or Yemen* or Zambia* or Zimbabwe* or Russia* or Yugoslavia* or USSR or Soviet* or Byelarus*)<br>11 TS=(africa* or asia* or caribbean or "central america*" or "latin america*" or "south america*" or melanesia* or micronesia* or polynesia*)<br>12 TS=(“resource-limit*” or “resource-poor” or “low-resource*” or “limited-resource*” or “resource-constrain*” or “constrain*-resource*” or “under-resource*” or “poor*-resource*” or “resource-scarce*” or “scarce*-resource*” or “low-income” or “middle-income” or lowincome or middleincome or (low* NEAR/2 “middle-income”))<br>13 TS=(((developing or underdeveloped or under-developed or emerging or "less-developed" or "least-developed" or "less-economically developed" or "least-economically developed" or "less-affluent" or "least-affluent" or deprived or poor) NEAR/0 (country or countries or nation or region or economy or economies)) or ((developing or underdeveloped or "under-developed" or "less-developed" or "least-developed") NEAR/0 (population* or world)) or ("third-world*" or thirdworld* or "3rd-world*" or lmic or lmics or "lami countr*" or "lalmi countr*" or "transitional countr*") or (low* NEAR/0/0(gdp or gnp or "gross domestic" or "gross national")) or ((underserved or "under-served") NEAR/0 (countr* or nation? or population*)))<br>14 #10 OR #11 OR #12 OR #13	12 TITLE-ABS-KEY ("resource-limit*" OR "resource-poor" OR "low-resource*" OR "limited-resource*" OR "resource-constrain*" OR "constrain*-resource*" OR "under-resource*" OR "poor*-resource*" OR "resource-scarce*" OR "scarce*-resource*" OR "low-income" OR "middle-income" OR lowincome OR middleincome OR (low* W/2 "middle-income"))<br>13 (TITLE-ABS-KEY ((developing OR underdeveloped OR "under-developed" OR emerging OR "less-developed" OR "least-developed" OR "less-economically developed" OR "least-economically developed" OR "less-affluent" OR "least-affluent" OR deprived OR poor) W/0 (country OR countries OR nation? OR region? OR economy OR economies))) OR (TITLE-ABS-KEY ((developing OR underdeveloped OR "under-developed" OR "less-developed" OR "least-developed") W/0 (population* OR world))) OR (TITLE-ABS-KEY ("third-world*" OR thirdworld* OR "3rd-world*" OR lmic OR lmics OR "lami countr*" OR "lalmi countr*" OR "transitional countr*")) OR (TITLE-ABS-KEY (low* W/0 (gdp OR gnp OR "gross domestic" OR "gross national") OR (underserved OR "under-served") W/0 (countr* OR nation? OR population<br>14 10 OR 11 OR 12 OR 13<br>15 3 OR 9 OR

resource*" or "under-resource*" or "poor*-resource*" or "resource-scarce*" or "scarce*-resource*" or "low-income" or "middle-income" or lowincome or middleincome or (low* N3 "middle-income")<br>S19 (((developing or underdeveloped or "under-developed" or emerging or "less-developed" or "least-developed" or "less-economically developed" or "least-economically developed" or "less-affluent" or "least-affluent" or deprived or poor) N0 (country or countries or nation? or region? or economy or economies)) or ((developing or underdeveloped or "under-developed" or "less-developed" or "least-developed") N0 (population* or world)) or ("third-world*" or thirdworld* or "3rd-world*" or lmic or lmics or "lami countr*" or "lalmi countr*" or "transitional countr*") or (low* N0 (gdp or gnp or "gross domestic" or "gross national")) or ((underserved or "under-served") N0 (countr* or nation? or population*)))<br>S20 S15 OR S16 OR S17 OR S18 OR S19<br>S21 S5 AND S14 AND S20	15 #3 AND #9 AND #14	

Appendix S2: Operational definitions of neurodevelopmental outcomes

1. Neurodevelopmental impairment: presence of any of the following - cerebral palsy, visual impairment/blindness, hearing impairment/deafness, development delays, or however defined by the authors.
2. Cerebral palsy: abnormality of tone with motor dysfunction diagnosed through clinical/neurological examination, or however defined by the authors.
3. Motor impairments (excluding CP): serious impairment in the development of motor coordination, assessed using validated method/assessment tool, or however defined by the authors. (e.g. developmental coordination disorder)
4. Developmental delays:
    a. Motor, Cognitive or language delays: a score of less than -1SD below population mean, assessed using validated method/assessment tool, or however defined by the authors.
    b. Global developmental delays: presence of delay in two or more developmental domains (e.g. motor, cognitive, language)
5. Hearing impairment including deafness: hearing loss requiring amplification or worse
6. Vision impairment including blindness: severe visual impairment with a visual acuity of <3/60 in either eye assessed by standard method.
7. Learning difficulties**:** however, defined by the authors using age-appropriate assessment tool
8. Adverse behavioural and social-emotional outcomes: includes screening or clinical diagnosis of Autism spectrum disorder (ASD), Attention-deficit/hyperactivity disorder (ADHD), externalizing and internalizing behavioral problems.
9. Growth outcomes:
    a. Undernutrition: weight for age z score <2SD
    b. Stunting: height for age z score <2SD

Figure S1: Prevalence of neurodevelopmental impairment

Study	Country	GA (wks)	Age at f/up	Number of cases	Total participant	Proportion with 95% CI	Weight (%)
Li 2019	China	<28	18-24 mo	47	131	0.36 [0.28, 0.44]	8.61
Wang 2022	China	<30	18-24 mo CA	113	709	0.16 [0.13, 0.19]	9.65
Chen 2021	China	<32	1y CA	107	1,092	0.10 [0.08, 0.12]	9.75
Xue 2016	China	≤32	1y CA	22	105	0.21 [0.14, 0.29]	8.33
Eras 2013	Turkey	≤32	12-18mo CA	85	370	0.23 [0.19, 0.27]	9.41
Bozkurt 2017	Turkey	≤32	18-22mo CA	46	220	0.21 [0.16, 0.27]	9.09
Sivanandan 2022	India	≤32	18 mo CA	25	195	0.13 [0.08, 0.18]	9.00
Sujatha 2016	India	≤33	1y CA	14	225	0.06 [0.03, 0.10]	9.11
Airede 1992	Nigeria	27-34	2y CA	10	159	0.06 [0.03, 0.11]	8.81
Saha 2023	India	34-36	2y CA	35	299	0.12 [0.08, 0.16]	9.30
Do 2020	Vietnam	<37	2y CA	43	183	0.23 [0.18, 0.30]	8.94
**Overall**						0.16 [0.11, 0.21]	

Heterogeneity: $\tau^2 = 0.05$, $I^2 = 93.91\%$, $H^2 = 16.42$

Test of $\theta_i = \theta_j$: $Q(10) = 124.62$, $p = 0.00$

Test of $\theta = 0$: $z = 10.83$, $p = 0.00$

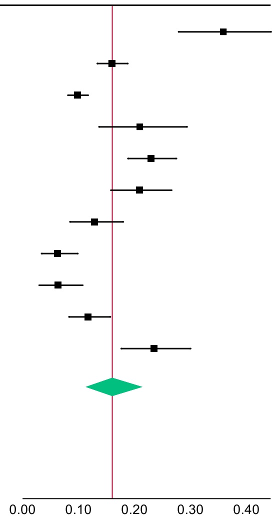

Figure S2: Prevalence of cerebral palsy

Study	Country	GA (wks)	Age at f/u	Number of cases	Total participant	Proportion with 95% CI	Weight (%)
Li 2019	China	<28	18-24mo	14	131	0.11 [0.06, 0.17]	5.91
Wang 2022	China	<30	18-24mo CA	29	709	0.04 [0.03, 0.06]	8.14
Chen 2021	China	<32	1y CA	22	1,092	0.02 [0.01, 0.03]	8.39
Gocer 2011	Turkey	≤32	35.8mo	10	117	0.09 [0.04, 0.14]	5.68
Bozkurt 2017	Turkey	≤32	18-22 mo CA	14	220	0.06 [0.03, 0.10]	6.84
Eras 2013	Turkey	≤32	12-18mo	14	370	0.04 [0.02, 0.06]	7.55
Sivanandan 2022	India	≤32	18mo CA	5	195	0.03 [0.01, 0.05]	6.64
Luo 2013	China	≤34	12 mo CA	11	129	0.09 [0.04, 0.14]	5.88
Burger 2011	South Africa	<37	1y CA	9	115	0.08 [0.04, 0.14]	5.64
Were 2006	Kenya	<37	2y CA	14	120	0.12 [0.06, 0.18]	5.73
Do 2020	Vietnam	<37	2y CA	13	181	0.07 [0.04, 0.11]	6.51
Li 2001	China	<37	1-6y	290	8,256	0.04 [0.03, 0.04]	8.84
Saha 2023	India	34-36	2y CA	6	299	0.02 [0.01, 0.04]	7.28
You 2019a	China	34-36	24-30mo	2	102	0.02 [0.00, 0.06]	5.39
You 2019b	China	34-36	2-2.5y	2	112	0.02 [0.00, 0.05]	5.59
**Overall**						0.05 [0.03, 0.06]	

Heterogeneity: $\tau^2 = 0.01$, $I^2 = 87.20\%$, $H^2 = 7.81$

Test of $\theta_i = \theta_j$: $Q(14) = 64.44$, $p = 0.00$

Test of $\theta = 0$: $z = 10.35$, $p = 0.00$

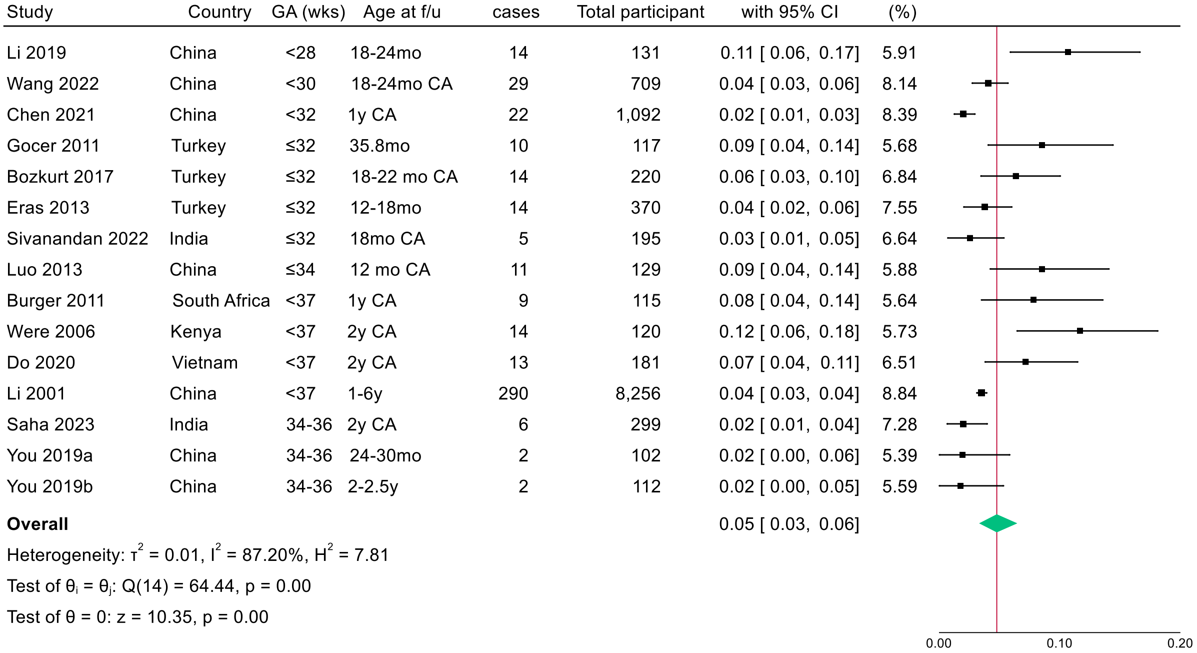

Figure S3: Prevalence of hearing impairment/deafness

Study	Country	GA (wks)	Age at f/up	Number of cases	Total participants	Proportion with 95% CI	Weight (%)
Wang 2022	China	<32	18-24mo CA	17	709	0.02 [0.01, 0.04]	17.22
Chen 2021	China	<32	1y CA	13	1,092	0.01 [0.01, 0.02]	18.17
Eras 2013	Turkey	≤32	12-18mo CA	0	370	0.00 [0.00, 0.00]	15.16
Sivanandan 2022	India	≤32	18mo CA	0	195	0.00 [0.00, 0.01]	12.38
Sujatha 2016	India	≤33	1y CA	1	225	0.00 [0.00, 0.02]	13.05
Song 2023	China	<34	4.0±1.5y	1	114	0.01 [0.01, 0.04]	9.71
Saha 2023	India	34-36	2y CA	6	299	0.02 [0.01, 0.04]	14.31
**Overall**						0.01 [0.00, 0.02]	

Heterogeneity: $\tau^2 = 0.01$, $I^2 = 75.82\%$, $H^2 = 4.14$

Test of $\theta_i = \theta_j$: Q(6) = 23.67, p = 0.00

Test of $\theta = 0$: z = 3.34, p = 0.00

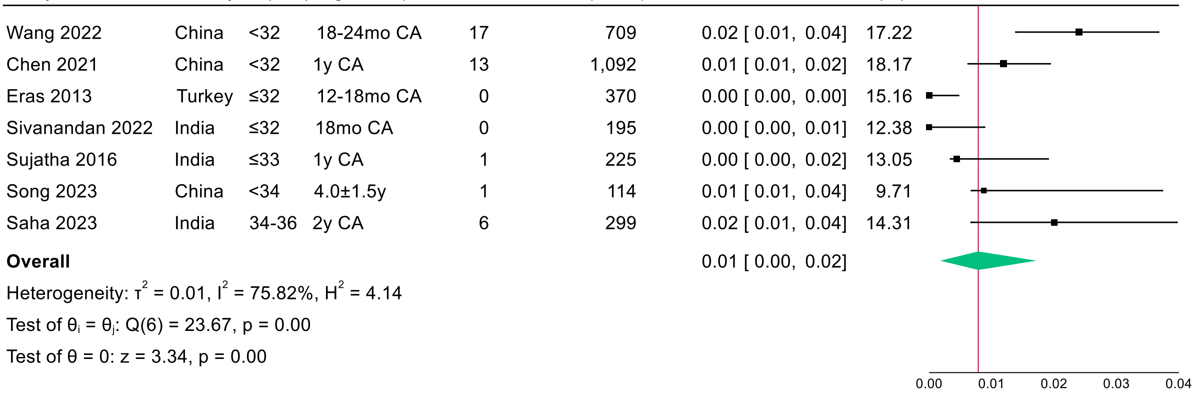

Figure S4: Prevalence of visual impairment/blindness

Study	Country	GA		Number of cases	Total participants	Proportion with 95% CI	Weight (%)
Wang 2022	China	<32	18-24mo CA	20	709	0.03 [0.02, 0.04]	15.86
Chen 2021	China	<32	1y CA	13	1,092	0.01 [0.01, 0.02]	16.79
Eras 2013	Turkey	≤32	12-18mo CA	0	370	0.00 [0.00, 0.00]	13.84
Gocer 2011	Turkey	≤32	35.8±2.3mo	2	117	0.02 [0.00, 0.05]	8.80
Sivanandan 2022	India	≤32	18mo CA	4	195	0.02 [0.00, 0.05]	11.18
Sujatha 2016	India	≤33	1y CA	1	225	0.00 [0.00, 0.02]	11.82
Song 2023	China	<34	4.0±1.5y	0	114	0.00 [0.00, 0.02]	8.68
Saha 2023	India	34-36	2y CA	4	299	0.01 [0.00, 0.03]	13.02
**Overall**						0.01 [0.00, 0.02]	

Heterogeneity: $\tau^2 = 0.01$, $I^2 = 72.47\%$, $H^2 = 3.63$

Test of $\theta_i = \theta_j$: Q(7) = 25.40, p = 0.00

Test of $\theta = 0$: z = 4.03, p = 0.00

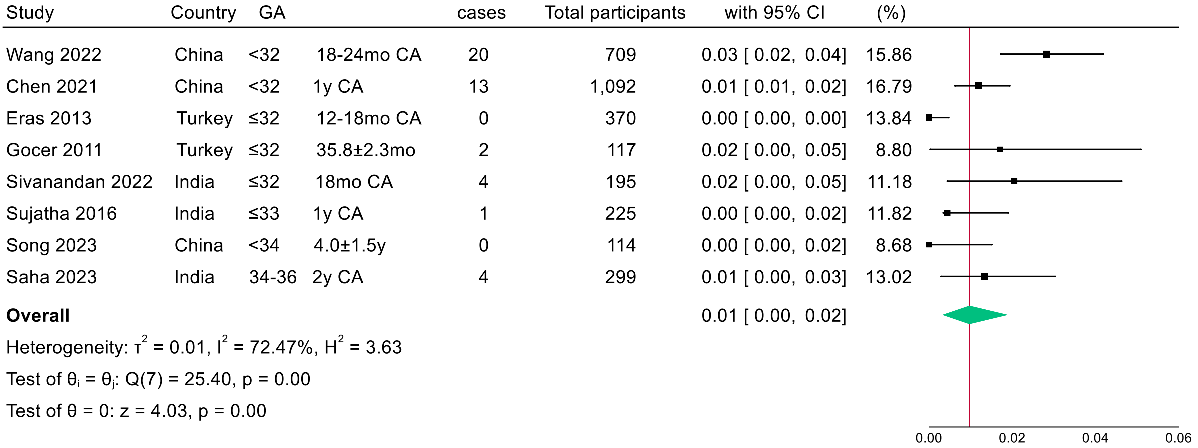

Figure S5: Prevalence of motor impairment

Study	Country	GA(wk)	Age at f/up	Tool used	Cutoff	Number of cases	Total participants	Proportion with 95% CI	Weight (%)
Liu 2023	China	<37	3-10y	MABC-2	≤5th percentile	17	156	0.11 [0.06, 0.16]	44.33
Du 2020	China	<37	3-10y	MABC-2	≤5th percentile	28	196	0.14 [0.10, 0.20]	55.67
**Overall**								0.13 [0.09, 0.16]	

Heterogeneity: $\tau^2 = 0.00$, $I^2 = 0.00\%$, $H^2 = 1.00$

Test of $\theta_i = \theta_j$: Q(1) = 0.86, p = 0.35

Test of $\theta = 0$: z = 12.41, p = 0.00

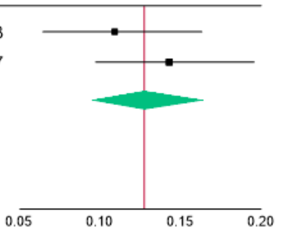

Figure S6: Prevalence of motor delays

Study	Country	GA(wks)	Age at f/up	Assessment tool	Cutoff used	Number of cases	Total participants	Proportion with 95% CI	Weight (%)
**Overall/any motor delay**									
Bozkurt 2017	Turkey	≤32	18-22mo CA	BSID-II	<70	31	220	0.14 [0.10, 0.19]	4.26
Eras 2013	Turkey	≤32	12-18mo CA	BSID-II	<70	71	370	0.19 [0.15, 0.23]	4.36
Sivanandan 2022	India	≤32	18mo CA	DASII	<70	18	182	0.10 [0.06, 0.15]	4.21
Luo 2013	China	27-34	12mo CA	PDMS-2	<80	28	129	0.22 [0.15, 0.29]	4.10
Moreira 2014	Brazil	<35	8-10y	MABC-2	<15thpercentile	39	100	0.39 [0.30, 0.49]	3.99
Akshara 2020	India	32-36	1y	TDSC+DASII	NR	53	158	0.34 [0.26, 0.41]	4.17
Ferreira 2014	Brazil	<37	12mo CA	BSID-II+clinical	<85	79	194	0.41 [0.34, 0.48]	4.23
Mello 2009	Brazil	<37	12mo CA	BSID-II	<85	26	100	0.26 [0.18, 0.35]	3.99
Do 2020	Vietnam	<37	24mo CA	BSID-III	<-2SD to <-1SD	31	180	0.17 [0.12, 0.23]	4.21
You 2019a	China	34-36	24-30mo	GDDS	<75	7	102	0.07 [0.03, 0.13]	4.00
You 2019b	China	34-36	2-2.5y	GDDS	<75	9	112	0.08 [0.04, 0.14]	4.04
Saha 2023	India	34-36	2y CA	DASII	<70	26	299	0.09 [0.06, 0.12]	4.33
Heterogeneity: τ^2 = 0.08, I^2 = 93.47%, H^2 = 15.31								0.19 [0.13, 0.26]	
Test of $\theta_i = \theta_j$: Q(11) = 153.55, p = 0.00									
Test of θ = 0: z = 9.71, p = 0.00									
**Gross motor delay**									
Gocer 2011	Turkey	≤32	35.8mo	DDST II	NR	12	117	0.10 [0.05, 0.16]	4.06
Ratanatharathorn 2022	Thailand	<37	12mo CA	BSID-III	<7	12	126	0.10 [0.05, 0.15]	4.09
Mackay 2022	South Africa	<37	12mo PMA	GMDS III	<-2SD to –1SD	2	104	0.02 [0.00, 0.06]	4.01
Hua 2022	China	<37	3-5y	ASQ-3	<2SD	3,147	17,753	0.18 [0.17, 0.18]	4.52
Bao 2023	China	<37	12mo	NDSC	<85	21	144	0.15 [0.09, 0.21]	4.14
Metwally 2023	Egypt	<37	6-12y	VABS	<70	10	186	0.05 [0.03, 0.09]	4.22
Heterogeneity: τ^2 = 0.04, I^2 = 90.77%, H^2 = 10.84								0.09 [0.05, 0.15]	
Test of $\theta_i = \theta_j$: Q(5) = 71.93, p = 0.00									
Test of θ = 0: z = 6.53, p = 0.00									
**Fine motor delay**									
Gocer 2011	Turkey	≤32	35.8mo	DDST II	NR	15	117	0.13 [0.07, 0.20]	4.06
Burger 2011	South Africa	<37	12mo CA	PDMS-2/AIMS		5	115	0.04 [0.01, 0.09]	4.05
Ratanatharathorn 2022	Thailand	<37	12mo CA	BSID-III	<7	7	126	0.06 [0.02, 0.10]	4.09
Hua 2022	China	<37	3-5y	ASQ-3	<2SD	963	17,753	0.05 [0.05, 0.06]	4.52
Bao 2023	China	<37	12mo	NDSC	<85	23	144	0.16 [0.10, 0.22]	4.14
Metwally 2023	Egypt	<37	6-12y	VABS	<70	13	186	0.07 [0.04, 0.11]	4.22
Heterogeneity: τ^2 = 0.02, I^2 = 83.52%, H^2 = 6.07								0.08 [0.05, 0.12]	
Test of $\theta_i = \theta_j$: Q(5) = 27.90, p = 0.00									
Test of θ = 0: z = 7.81, p = 0.00									
**Overall**								0.13 [0.10, 0.18]	
Heterogeneity: τ^2 = 0.07, I^2 = 98.67%, H^2 = 75.02									
Test of $\theta_i = \theta_j$: Q(23) = 1709.34, p = 0.00									
Test of θ = 0: z = 11.69, p = 0.00									
Test of group differences: $Q_b(2)$ = 10.12, p = 0.01									

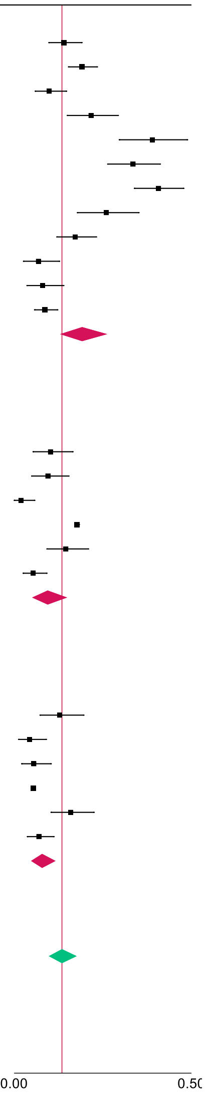

Figure S7: Prevalence of cognitive delays

Study	Country	GA(wks)	Age at f/up	Assessement tool	Cutoff used	Number of cases	Total participant	Proportion with 95% CI	Weight (%)
Wang 2022	China	<30	18-24 mo CA	BSID-II	<70	76	709	0.11 [0.09, 0.13]	12.80
Eras 2013	Turkey	≤32	12-18mo CA	BSID-II	<70	45	370	0.12 [0.09, 0.16]	12.67
Bozkurt 2017	Turkey	≤32	18-22mo CA	BSID-II	<70	24	220	0.11 [0.07, 0.15]	12.50
Sivanandan 2022	India	≤32	18mo CA	DASII	<70	5	182	0.03 [0.01, 0.06]	12.41
Were 2006	Kenya	<37	24 mo CA	Dorothy Egan's model	NR	11	120	0.09 [0.05, 0.15]	12.16
Ferreira 2014	Brazil	<37	12mo CA	BSID-II	<85	76	194	0.39 [0.32, 0.46]	12.44
Do 2020	Vietnam	<37	24mo CA	BSID-III	<-1SDto<-2SD	77	184	0.42 [0.35, 0.49]	12.42
Saha 2023	India	34-36	2y CA	DASII	<85	41	299	0.14 [0.10, 0.18]	12.61
**Overall**								0.16 [0.08, 0.26]	
Heterogeneity: τ^2 = 0.13, I^2 = 97.21%, H^2 = 35.87									
Test of $\theta_i = \theta_j$: Q(7) = 186.28, p = 0.00									
Test of θ = 0: z = 5.82, p = 0.00									

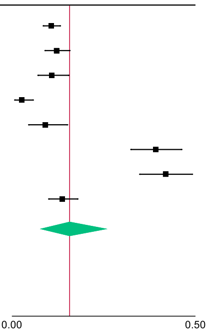

Figure S8: Prevalence of language delay

Study	Country	GA (wk)	Tool used	Number of cases	Total participant	Proportion with 95% CI	Weight (%)
Li 2019	China	<28	S-S relations	6	131	0.05 [0.02, 0.09]	19.88
Gocer 2011	Turkey	≤32	DDST II	27	117	0.23 [0.16, 0.31]	19.55
Do 2020	Vietnam	<37	BSID III	14	179	0.08 [0.04, 0.12]	20.66
Ratanatharathorn 2022	Thailand	<37	BSID III	12	126	0.10 [0.05, 0.15]	19.77
Bao 2023	China	<37	NDSC	27	144	0.19 [0.13, 0.26]	20.14
**Overall**						0.12 [0.06, 0.19]	

Heterogeneity: $\tau^2 = 0.05$, $I^2 = 86.55\%$, $H^2 = 7.44$

Test of $\theta_i = \theta_j$: Q(4) = 28.63, p = 0.00

Test of $\theta = 0$: z = 6.06, p = 0.00

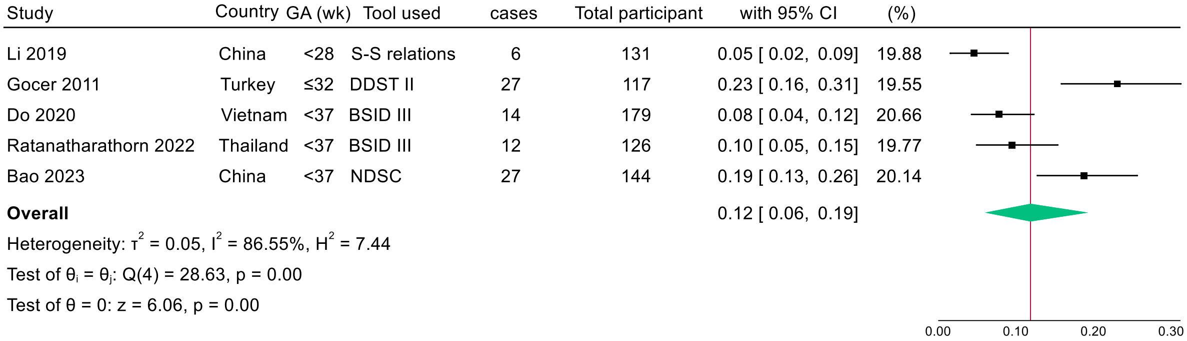

Figure S9: Prevalence of global developmental delay

Study	Country	GA(wk)	Age at f/up	Assessement tool	Number of cases	Total participant	Proportion with 95% CI	Weight (%)
Li 2019	China	<28	18-24mo	S-S relations+GDS	14	131	0.11 [0.06, 0.17]	23.08
Eras 2013	Turkey	≤32	12-18mo CA	BSID II	30	370	0.08 [0.06, 0.11]	26.62
Sivanandan 2022	India	≤32	18mo CA	DASII	5	182	0.03 [0.01, 0.06]	24.49
Baskabadi 2016	Iran	<37	24mo CA	ASQ	39	270	0.14 [0.10, 0.19]	25.81
**Overall**							0.08 [0.04, 0.14]	

Heterogeneity: $\tau^2 = 0.03$, $I^2 = 86.93\%$, $H^2 = 7.65$

Test of $\theta_i = \theta_j$: Q(3) = 21.51, p = 0.00

Test of $\theta = 0$: z = 5.73, p = 0.00

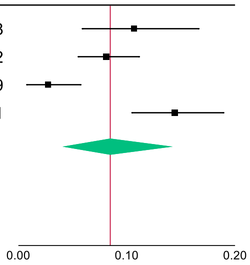

Figure S10: Prevalence of ADHD

Study	Country	GA(wks)	Age at f/up	Number of cases	Total participants	Proportion with 95% CI	Weight (%)
Shen 2022	China	<37	6-12y	70	1,055	0.07 [0.05, 0.08]	51.33
Soldateli 2022	Brazil	<37	6y	11	472	0.02 [0.01, 0.04]	48.67
**Overall**						0.04 [0.01, 0.09]	

Heterogeneity: $\tau^2 = 0.02$, $I^2 = 93.03\%$, $H^2 = 14.34$

Test of $\theta_i = \theta_j$: Q(1) = 14.34, p = 0.00

Test of $\theta = 0$: z = 3.64, p = 0.00

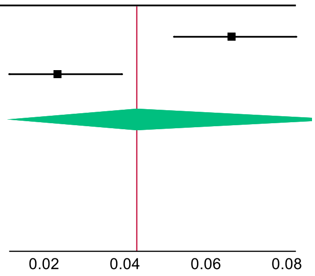

Figure S11: Prevalence of underweight

Study	Country	GA(wks)	Age at f/up	Number of cases	Total participants	Proportion with 95% CI	Weight (%)
Sivanandan 2022	India	≤32	18mo CA	75	194	0.39 [0.32, 0.46]	19.84
Do 2021	Vietnam	<37	24mo	10	182	0.05 [0.03, 0.09]	19.80
Liu 2019	China	<37	12mo	14	309	0.05 [0.02, 0.07]	20.05
Xiong 2014	China	<37	9-15y	8	226	0.04 [0.01, 0.06]	19.92
Yang 2010	China	<37	3.04-6.93y	187	8,923	0.02 [0.02, 0.02]	20.40
**Overall**						0.08 [0.01, 0.21]	

Heterogeneity: $\tau^2 = 0.18$, $I^2 = 98.71\%$, $H^2 = 77.42$

Test of $\theta_i = \theta_j$: $Q(4) = 221.26$, $p = 0.00$

Test of $\theta = 0$: $z = 2.76$, $p = 0.01$

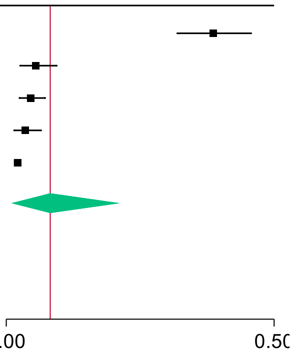

Figure S12: Prevalence of stunting

Study	Country	GA(wks)	Age at f/up	Number of cases	Total participants	Proportion with 95% CI	Weight (%)
Sivanandan 2022	India	≤32	18mo CA	36	194	0.19 [0.13, 0.24]	19.71
Do 2021	Vietnam	<37	24mo	25	182	0.14 [0.09, 0.19]	19.64
Liu 2019	China	<37	12mo	38	309	0.12 [0.09, 0.16]	20.08
Xiong 2014	China	<37	9-15y	0	226	0.00 [0.00, 0.01]	19.85
Yang 2010	China	<37	3.04-6.93 y	857	8,923	0.10 [0.09, 0.10]	20.72
**Overall**						0.09 [0.03, 0.19]	

Heterogeneity: $\tau^2 = 0.10$, $I^2 = 97.68\%$, $H^2 = 43.01$

Test of $\theta_i = \theta_j$: $Q(4) = 91.39$, $p = 0.00$

Test of $\theta = 0$: $z = 3.89$, $p = 0.00$

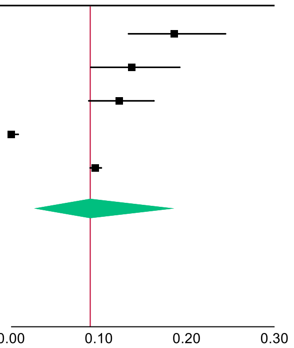

Figure S13: Funnel plots

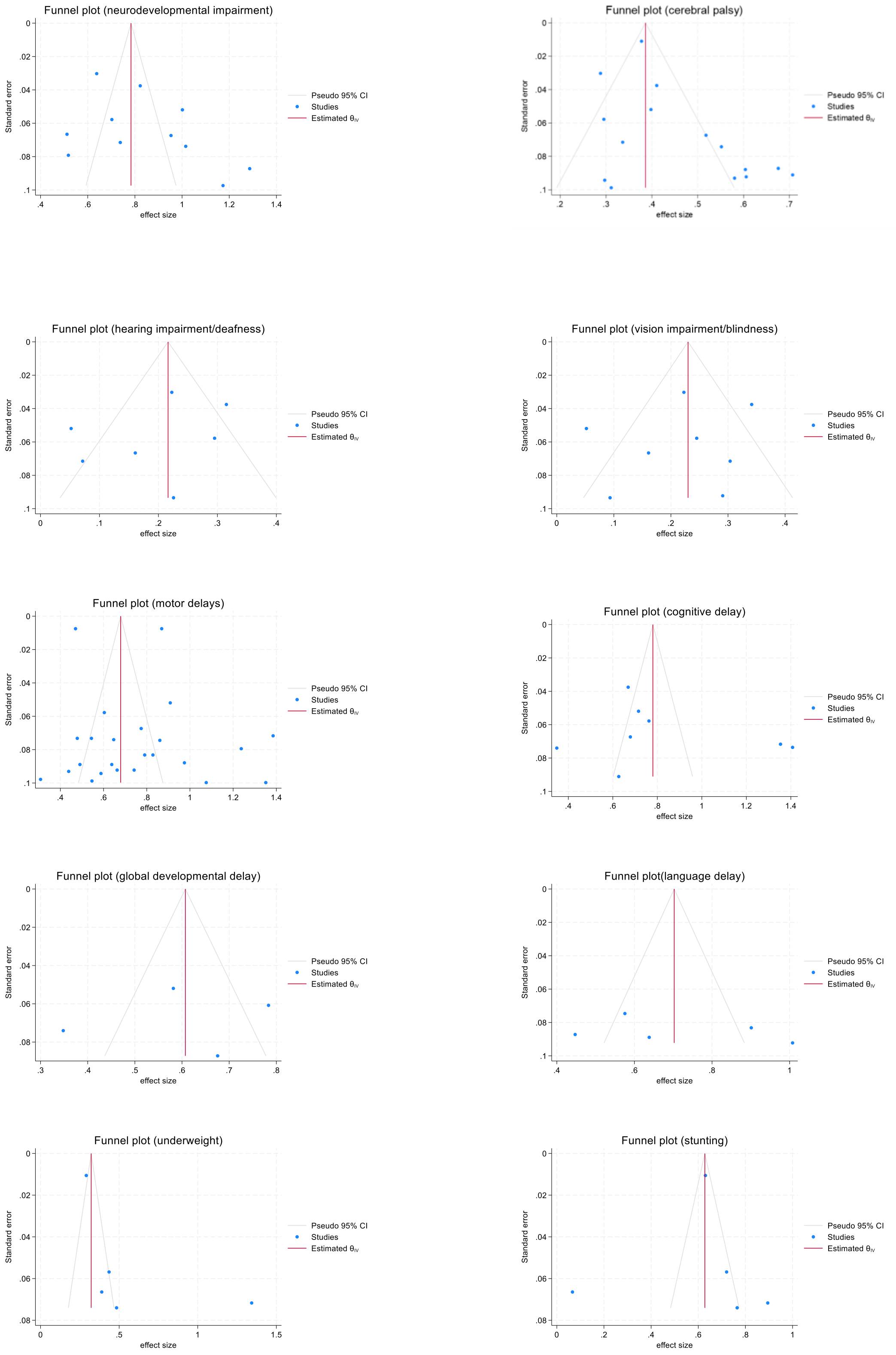

Table S1: Subgroup analysis by mean gestational age

Subgroup	Number of studies	Number of cases	Total participants	Prevalence (%)	95% CI	I^2(%)	Test of group difference
**Neurodevelopmental impairment**							
<28 weeks	1	47	131	36	28 – 44	-	0.00
28 to <32 weeks	9	465	3258	15	10 – 20	92.18	
32 to 36 weeks	1	35	299	12	8 – 16	-	
**Cerebral palsy**							
<28 weeks	1	14	131	11	6 – 17	-	0.06
28 to <32 weeks	7	92	2882	4	3 – 6	75.02	
32 to 36 weeks	4	24	633	4	1 – 8	82.63	
Not reported	3	311	8502	6	3 – 11	79.98	
**Visual impairment/blindness**							
<28 weeks	NA						0.66
28 to <32 weeks	6	38	2705	1	0 – 2	80.43	
32 to 36 weeks	1	4	299	1	0 – 3	-	
Not reported	1	2	117	2	0 – 5	-	
**Hearing impairment/deafness**							
<28 weeks	NA						0.11
28 to <32 weeks	6	32	2705	1	0 - 2	76.83	
32 to 36 weeks	1	6	299	2	1 - 4	-	
**Motor delays**							
<28 weeks	NA						0.04
28 to <32 weeks	10	282	1717	13	7 - 21	94.16	
32 to 36 weeks	3	42	513	8	6 - 11	0.00	
Not reported	11	4324	36787	15	10 – 22	99.33	
**Cognitive delays**							
<28 weeks	NA						0.42
28 to <32 weeks	6	302	1869	17	7- 32	98.03	
32 to 36 weeks	2	52	419	12	8-16	36.63	
**Global developmental delay**							
<28 weeks	1	14	131	11	6 – 17	-	0.50
28 to <32 weeks	3	74	822	8	3 – 16	91.75	

32 to 36 weeks	NA						
**Language delay**							
<28 weeks	1	6	131	5	2 – 9	-	0.00
28 to <32 weeks	2	26	305	8	6 – 12	0.00	
32 to 36 weeks	NA						
Not reported	2	54	261	21	16 – 26	0.00	

Table S2: Subgroup analysis by mean birthweight

Subgroup	Number of studies	Number of cases	Total participants	Prevalence (%)	95% CI	I^2(%)	Test of group difference
**Neurodevelopmental impairment**							
<1500 gm	8	455	2981	17	12 – 24	94.36	0.00
1500 to <2500 gm	2	78	482	17	7 – 30	91.04	
Not reported	1	14	225	6	3 – 10	-	
**Cerebral palsy**							
<1500 gm	8	121	2952	5	3 – 8	85.82	0.17
1500 to <2500 gm	2	19	480	4	1 – 11	86.53	
≥2500 gm	2	4	214	2	0 – 4	0.00	
Not reported	3	311	8502	6	3 -11	79.98	
**Visual impairment/blindness**							
<1500 gm	4	37	2366	1	0 – 3	87.94	0.91
1500 to <2500 gm	1	4	413	1	0 – 2	47.61	
Not reported	2	3	342	1	0 – 2	23.30	
**Hearing impairment/deafness**							
<1500 gm	4	30	2366	1	0 - 2	87.66	0.27
1500 to <2500 gm	2	7	299	2	1 - 3	0.00	
Not reported	NA	1	225	0	0 – 2	-	
**Motor delays**							
<1500 gm	11	278	1771	13	7 – 19	93.09	0.82
1500 to <2500 gm	2	57	479	13	5 – 22	85.57	
≥2500 gm	4	60	502	11	7 – 16	59.66	
Not reported	7	4253	36265	17	8 – 28	99.75	
**Cognitive delays**							
<1500 gm	6	237	1795	13	5 – 23	96.26	0.32
1500 to <2500 gm	2	118	483	26	5 - 57	97.90	
≥2500 gm	NA						
**Global developmental delay**							
<1500 gm	3	49	683	7	3 – 12	81.52	0.03
1500 to <2500 gm	1	39	270	14	10 – 19	-	
≥2500 gm	NA						
**Language delay**							

<1500 gm	3	45	374	11	3 – 24	90.11	0.01
1500 to <2500 gm	1	14	179	8	4 – 12	-	
≥2500 gm	1	27	144	19	13 – 26	-	

Table S3: Subgroup analysis by country-income level

Subgroup	Number of studies	Number of cases	Total participants	Prevalence (%)	95% CI	I^2(%)	Test of group difference
**Neurodevelopmental impairment**							
Upper-middle - income	6	420	2627	20	14 – 27	94.07	0.06
Lower-middle-income	5	127	1061	11	6 – 18	88.45	
**Cerebral palsy**							
Upper-middle - income	11	417	11353	5	3 – 7	86.75	0.87
Lower-middle-income	4	38	795	5	2 -10	85.35	
**Visual impairment/blindness**							
Upper-middle - income	5	35	2402	1	0 – 1	83.63	0.66
Lower-middle-income	3	9	719	1	0 – 2	0.00	
**Hearing impairment/deafness**							
Upper-middle - income	4	31	114	1	0 – 2	83.93	0.78
Lower-middle-income	3	7	719	1	0 – 2	67.49	
**Motor delays**							
Upper-middle - income	14	4497	37826	14	9 – 19	99.00	0.75
Lower-middle-income	5	151	1191	12	6 – 21	93.45	
**Cognitive delays**							
Upper-middle - income	5	282	1792	16	8 – 27	96.37	0.91
Lower-middle-income	3	93	486	15	0 – 42	97.94	
**Global developmental delay**							
Upper-middle - income	2	44	501	9	6 – 11	0.00	0.86
Lower-middle-income	2	44	452	8	0 – 23	95.16	
**Language delay**							
Upper-middle - income	4	72	518	13	6 – 23	87.99	0.24
Lower-middle-income	1	14	179	8	4 – 12	-	
**Underweight**							
Upper-middle - income	3	209	9458	3	2 – 5	71.94	0.20
Lower-middle-income	2	85	376	19	0 – 59	98.56	
**Stunting**							

Upper-middle - income	3	895	9458	5	0 – 18	98.41	0.09
Lower-middle-income	2	61	376	16	12 – 21	36.92	

Table S4: Sensitivity analysis for high quality studies

**Outcome**	**Number of studies**	**Pooled estimate of prevalence (%, 95% CI)**	**I^2 (%)**
Neurodevelopmental impairment	9	15 (10 -21)	95.24
Cerebral palsy	10	5 (3 – 7)	84.67
Visual impairment/blindness	7	1 (0 – 2)	76.36
Motor delays	13	12 (7 – 17)	99.32
Cognitive delays	7	16 (7 – 29)	97.60
Global developmental delays	3	7 (3 – 12)	81.52
Language delay	3	11 (3 – 23)	91.56
